# Relationship between gestational diabetes mellitus and anxiety symptoms and gut microbiome composition in pregnant women

**DOI:** 10.1515/biol-2025-1317

**Published:** 2026-05-25

**Authors:** Jing Xu, Lina Cai, Sushuang Lu, Lina Chen, Tiantian Zhang, Jing Wang

**Affiliations:** Department of Obstetrics and Gynecology, First Hospital of Hebei Medical University, Shijiazhuang 050000, China; Graduate School, Hebei Medical University, Shijiazhuang 050000, China

**Keywords:** gestational diabetes mellitus (GDM), anxiety symptoms, gut-brain axis, gut microbiome

## Abstract

Gestational diabetes mellitus (GDM) is a common metabolic complication of pregnancy associated with poor outcomes for both mother and baby. An expanding body of research highlights the gut microbiota’s influence on host metabolism and neurobehavioral processes, yet how gestational diabetes mellitus, anxiety symptoms, and microbial communities interact is still unclear. To address this gap, we profiled gut-microbiome changes in pregnant women presenting with both GDM and clinically significant anxiety. A total of 120 participants were categorized into four groups: GDM with anxiety (G + A), GDM without anxiety (G + NAF), non-GDM with anxiety (NG + A), and non-GDM without anxiety (NG + NA). Stool specimens were obtained and subjected to 16S rRNA gene profiling of the V3–V4 hypervariable region using Illumina MiSeq sequencing. Results showed significant differences in fasting blood glucose between GDM and non-GDM groups. GDM groups exhibited enrichment of Enterobacteriaceae and reduction of Faecalibacterium, while anxiety groups showed increased Lactococcus and Streptococcus. The dual-exposure group (G + A) demonstrated unique enrichment of oral-derived genera (e.g., Fusobacterium and Actinomyces). Among 963 OTUs identified, 681 were core OTUs shared across groups. GDM and anxiety groups possessed 42 and 32 unique OTUs, respectively, with 26 OTUs specific to the G + A group. LEfSe analysis revealed 16 phylogenetically conserved taxa distinguishing GDM and non-GDM groups, while anxiety-positive groups were characterized by Bacilli and Negativicutes. These findings suggest that GDM and anxiety are associated with distinct gut microbiota profiles, highlighting potential microbial biomarkers for early diagnosis and intervention.

## 1 Background

Gestational diabetes mellitus (GDM) stands as a prevalent metabolic condition arising during pregnancy, marked by the onset of impaired glucose tolerance specifically in the gestational period. This disorder elevates the likelihood of maternal complications, including hypertension during pregnancy and an increased rate of cesarean sections. It also poses significant risks to the fetus and newborn, such as excessive birth weight (macrosomia), low blood sugar in the neonate, and respiratory difficulties [1], 2]. Furthermore, individuals with a history of GDM face a substantially greater lifelong risk of progressing to type 2 diabetes, and their children are more susceptible to developing metabolic syndromes later in life. Emerging research continues to underscore a strong link between the composition of gut microbiota and the metabolic health of the host, suggesting that intestinal bacteria play a crucial role in regulating metabolic processes, immune function, and hormonal balance [3], [4], [5].

Anxiety is a prevalent psychological concern during pregnancy, especially in individuals diagnosed with gestational diabetes mellitus (GDM). This condition not only compromises the mental health of the expectant mother but is also associated with potential risks to fetal development. Studies suggest that anxiety can stimulate the hypothalamic-pituitary-adrenal (HPA) axis, resulting in increased secretion of stress hormones (e.g., cortisol), which may subsequently alter the diversity and ecological balance of the gut microbiome [6], [7], [8]. Conversely, the gut microbiota can modulate the host’s emotional and behavioral responses through the synthesis of neuroactive compounds, including short-chain fatty acids and metabolites derived from tryptophan. This interplay implies a potential bidirectional communication pathway between the gut microbial community and the host’s psychological state [9], 10].

In recent years, a growing body of research has underscored significant associations between the composition of gut microbiota and various neurodevelopmental conditions, including autism spectrum disorder (ASD) [11], 12]. For example, investigations have revealed distinct gut microbial profiles in children with ASD, characterized by increased bacterial richness and the presence of specific microbial markers such as Alcaligenaceae and Acinetobacter, differences that are also observed, to some extent, in their mothers [13]. This suggests that maternal gut microbiota may play a role in offspring neurodevelopment, potentially through mechanisms of vertical transmission. Nevertheless, studies exploring the interplay between gestational diabetes mellitus (GDM), anxiety symptoms, and the gut microbial community in pregnant women remain limited. The precise causal pathways and molecular mechanisms underlying these interactions are yet to be thoroughly clarified.

The present study aims to investigate the correlation between gestational diabetes mellitus (GDM) and anxiety symptoms and the gut microbiota composition in pregnant women. We designed four experimental groups: A: GDM without anxiety (GDM + no anxiety, G + NA), B: GDM with anxiety (GDM + anxiety, G + A), C: no GDM without anxiety (No GDM + no anxiety, NG + NA), and D: no GDM with anxiety (No GDM + anxiety, NG + A). By analyzing the structure of gut microbial communities across these groups, we seek to elucidate whether alterations in gut microbiota composition are associated with the occurrence and progression of GDM as well as the presence of anxiety symptoms. Additionally, we aim to explore potential biomarkers within the gut microbiota that may provide novel insights and methodologies for the early diagnosis and intervention of GDM.

## 2 Materials and methods

### 2.1 Study population

Between June 2023 and October 2024, we recruited 120 pregnant women undergoing prenatal care at the Department of Obstetrics, First Hospital of Hebei Medical University, as subjects for this study. These women were divided into four groups, each comprising 40 individuals: gestational diabetes mellitus with anxiety (G + A), gestational diabetes mellitus without anxiety (G + NA), no gestational diabetes mellitus with anxiety (NG + A), and no gestational diabetes mellitus without anxiety (NG + NA). This study is an observational case-control study, which has been approved by the First Ethics Committee of Hebei Medical University (approval number [2024] Research Review No. S01228).

Inclusion criteria for the participants were as follows:

(1) Singleton primiparous women at 24–37 weeks of gestation;
(2) Adult patients aged ≥20 years;
(3) Singleton pregnancy;
(4) Conception via natural means;
(5) Complete clinical data;
(6) Consent to participate in the research.

Exclusion criteria for the participants were as follows:

(1) Type 1 diabetes mellitus;
(2) Gastrointestinal malignancies or prior gastrointestinal surgery;
(3) Prior colectomy;
(4) History of gastrointestinal diseases;
(5) Infections of the gastrointestinal, respiratory, or urinary tracts;
(6) History of antibiotic use in the preceding 2 months;
(7) Consumption of probiotics, laxatives, or antidiarrheal agents within the past 2 weeks;
(8) Pregnancy-induced hypertension or thyroid dysfunction during pregnancy;
(9) Pregnancy complicated by hepatitis;
(10) Pregnancy-associated cholestasis;
(11) Pregnancy in women with pre-existing heart disease;
(12) Pre-pregnancy type 2 diabetes mellitus or other relevant chronic diseases;
(13) Presence of psychiatric disorders that would preclude completion of the relevant surveys.

Participants’ anxiety levels in early pregnancy were gauged at enrollment through the Self-rating Anxiety Scale (SAS), capturing their self-reported emotional status. Anxiety in early pregnancy was evaluated based on the Chinese normative standards, with a SAS standard score ≥50 indicating the presence of anxiety.

**Informed consent:** Informed consent has been obtained from all individuals included in this study.

**Ethical approval:** The research related to human use has been complied with all the relevant national regulations, institutional policies and in accordance with the tenets of the Helsinki Declaration, and has been approved by the Ethics Committee of the First Hospital of Hebei Medical University (Approval number: [2023] Research Review No. S01228). Approval was also granted by The First Hospital of Hebei Medical University obstetrics and Gynecology Department to allow data collection on their premises.

### 2.2 Sample collection

Fecal specimens obtained from participants were preserved in sterile tubes with absolute ethanol and maintained at −80 °C until further processing. Genomic DNA was isolated from approximately 250 mg of each fecal sample employing the FastDNA SPIN Kit for Feces (MP Biomedicals, Santa Ana, CA, USA), adhering strictly to the supplier’s protocols. The concentration of the extracted DNA was then measured utilizing a NanoDrop ND-1000 spectrophotometer (Nucliber), with 1 μL allocated for each assessment.

### 2.3 DNA extraction and PCR amplification

For the DNA extraction procedure, the E.Z.N.A.^®^ Soil DNA Kit (Omega Bio-tek, Norcross, GA, USA) was applied following the manufacturer’s guidelines. The purity and concentration of the acquired DNA were verified with a NanoDrop 2000 spectrophotometer, while its integrity was inspected via 1 % agarose gel electrophoresis. Amplification of the bacterial 16S rRNA gene’s V3–V4 hypervariable regions was carried out using primers 338F (5′-ACT​CCT​ACG​GGA​GGC​AGC​AG-3′) and 806R (5′-GGACTACHVGGGTWTCTAAT-3′) for all DNA templates.

### 2.4 Illumina sequencing

After amplification, PCR amplicons were resolved on 2 % agarose, excised, and purified with the AxyPrep DNA Gel Extraction Kit (Axygen Biosciences), eluting in Tris-HCl. Concentrations were determined on a Qubit 4.0 fluorometer (Thermo Fisher). Libraries were constructed by ligating Y-adapters, removing dimers with magnetic beads, amplifying the target fragments, and denaturing into single-stranded DNA with NaOH. Sequencing was performed as 2 × 300 bp paired-end reads on an Illumina MiSeq, and the raw fastq files were uploaded to the NCBI Sequence Read Archive.

### 2.5 Data processing

Bioinformatic processing of the raw fastq data involved quality filtering using fastp software to eliminate adapter contamination and low-quality bases. Key steps included:

Truncating reads when the average quality within a 50-bp sliding window fell below 20.
Discarding sequences shorter than 50 bp after trimming.
Removing reads containing ambiguous nucleotides (N).
Merging paired-end reads permitting a maximum mismatch rate of 0.2 and a minimum overlap of 10 bp; unmerged reads were excluded.
Demultiplexing samples based on barcodes and primers, allowing zero mismatch in barcodes and up to two mismatches in primers.

Subsequently, sequence variants were delineated using Vsearch (v2.22.1) for clustering into operational taxonomic units (OTUs) at 97 % similarity, or using the DADA2 plugin in QIIME2 (v2022.8) for inferring amplicon sequence variants (ASVs). Taxonomic assignment of these OTU/ASV sequences was performed with the RDP Classifier (v2.13) against the Silva 138.1 database, applying a confidence threshold of 0.7.

### 2.6 Statistical analysis

Statistical analyses and data visualization were performed using the R programming environment, with the following packages utilized: vegan (v2.6-4), phyloseq (v1.38.0), tidyverse (v1.3.2), ggpubr (v0.5.0), ComplexHeatmap (v2.10.0), and corrplot (v0.92). Beta diversity was calculated using the Bray-Curtis dissimilarity index, and principal coordinates analysis (PCoA) as well as permutational multivariate analysis of variance (PERMANOVA) were conducted based on the resulting distance matrix. Non-parametric rank-sum tests were employed to detect differences in microbial communities between groups. Correlations between specific taxa were analyzed using Spearman’s rank correlation. The Benjamini-Hochberg (BH) method was applied for multiple testing correction of *p*-values, with a default significance level of 0.05.

## 3 Results

### 3.1 Clinical features of GDM and anxiety

**Figure 1:**
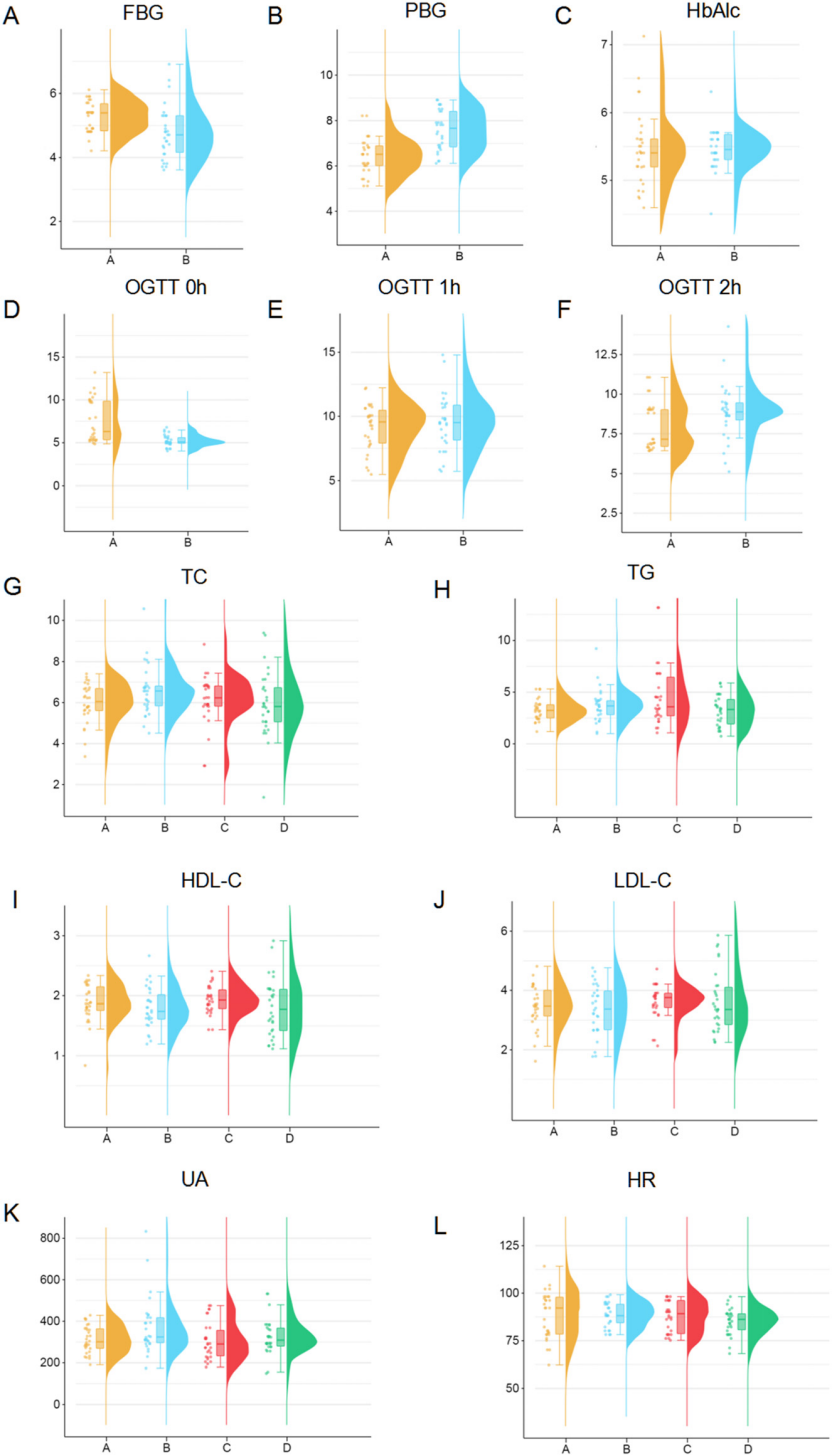
Clinical characterization analysis of four groups of samples. Clinical characteristics of patients across four groups. (A) Distribution of fasting blood glucose (FBG) levels. (B) Distribution of postprandial blood glucose (PBG) levels. (C) Distribution of glycated hemoglobin (HbA1c) levels. (D) Oral glucose tolerance test (OGTT) results at 0 h. (E): OGTT results at 1 h. (F): OGTT results at 2 h. (G) Total cholesterol (TC) levels. (H) Triglyceride (TG) levels. (I) High-density lipoprotein cholesterol (HDL-C) levels. (J) Low-density lipoprotein cholesterol (LDL-C) levels. (K) Serum uric acid (UA) levels. (L) Heart rate (HR) levels.

Based on the clinical characteristics of patients in terms of gestational diabetes mellitus (GDM) and anxiety, we collected clinical samples from four groups: Group A consisted of patients with GDM but without anxiety (GDM + no anxiety, G + NA), Group B included patients with GDM and anxiety (GDM + anxiety, G + A), Group C comprised patients without GDM or anxiety (No GDM + no anxiety, NG + NA), and Group D included patients with anxiety but without GDM (No GDM + anxiety, NG + A). We conducted a univariate analysis of the clinical data (Table 1) and compared the differences in clinical indicators among the different GDM and anxiety subgroups, with values presented as mean ± standard deviation (Figure 1). The results revealed a significant difference in fasting blood glucose (FBG) levels between the GDM and non-GDM groups (*p* < 0.05). Additionally, the FBG levels in the anxiety group were slightly higher than those in the non-anxiety group, although the difference did not reach statistical significance (*p* > 0.05), suggesting that anxiety may have a certain impact on blood glucose levels. In terms of lipid profiles, the GDM group exhibited significantly elevated total cholesterol and low-density lipoprotein cholesterol (LDL-C) levels, along with relatively lower high-density lipoprotein cholesterol (HDL-C) levels, which is consistent with the characteristic dyslipidemia often observed in GDM patients. However, no significant differences in lipid profiles were observed between the anxiety and non-anxiety groups (*p* > 0.05), indicating that anxiety has a limited effect on lipid metabolism. Significant differences were also observed in serum uric acid (UA) and glycated hemoglobin (HbA1c) levels between the GDM and non-GDM groups (*p* < 0.05). Although no significant difference in HbA1c levels was found between the anxiety and non-anxiety groups (*p* > 0.05), serum uric acid levels were slightly higher in the anxiety group, potentially related to metabolic stress under anxiety conditions. Heart rate showed a significant difference between the anxiety and non-anxiety groups (*p* < 0.05), and the anxiety scores in the GDM group were slightly higher than those in the non-GDM group, suggesting that GDM patients may face a higher risk of anxiety.

**Table 1:** Comparison of clinical characteristics of different gestational diabetes mellitus and anxiety groups.

Characteristics	A (GDM + no anxiety) (*n* = 30)	B (GDM + anxiety) (*n* = 30)	C (No GDM + no anxiety) (*n* = 30)	D (No GDM + anxiety) (*n* = 30)	*p*-Value
FBG	5.25 ± 0.47	4.76 ± 0.81	–	–	0.0065
PBG	6.42 ± 0.79	7.56 ± 0.86	–	–	0.00000212
TC	5.93 ± 0.94	6.55 ± 1.19	6.11 ± 1.20	5.92 ± 1.57	0.0452
TG	3.34 ± 1.01	3.67 ± 1.57	4.61 ± 2.93	3.19 ± 1.46	0.2059
HDL-C	1.87 ± 0.31	1.79 ± 0.34	1.92 ± 0.23	1.80 ± 0.47	0.3262
LDL-C	3.44 ± 0.70	3.35 ± 0.83	3.60 ± 0.57	3.60 ± 1.01	0.6649
UA	308.32 ± 64.77	370.28 ± 132.03	305.07 ± 88.48	323.31 ± 84.66	0.0315
HbAlc	5.46 ± 0.55	5.47 ± 0.30	–	–	0.9703
OGTT 0 h	7.47 ± 2.46	5.18 ± 0.67	–	–	0.0000094
OGTT 1 h	8.94 ± 1.76	9.51 ± 2.13	–	–	0.4562
OGTT 2 h	7.99 ± 1.47	8.84 ± 1.74	–	–	0.0505
HR	88.07 ± 12.04	88.50 ± 6.28	87.57 ± 7.78	84.80 ± 6.76	0.8641
Anxiety score	–	65.27 ± 5.92	–	57.03 ± 3.79	

### 3.2 OTU clustering and species composition analysis of the gut microbiome

In this microbiome study, a total of 15, 198, 187 high-quality 16S rRNA sequencing reads were generated. The median sequencing depth per sample reached 127,174 reads, with individual sample counts ranging from 83,111 to 176,657. After taxonomic classification, 963 operational taxonomic units (OTUs) were identified (see Supplementary Table S1). The sufficiency of sampling depth was corroborated by the species accumulation curve (Supplementary Figure S1), rarefaction analysis (Supplementary Figure S2), and rank abundance curves (Supplementary Figure S4) across all samples.

**Figure 2:**
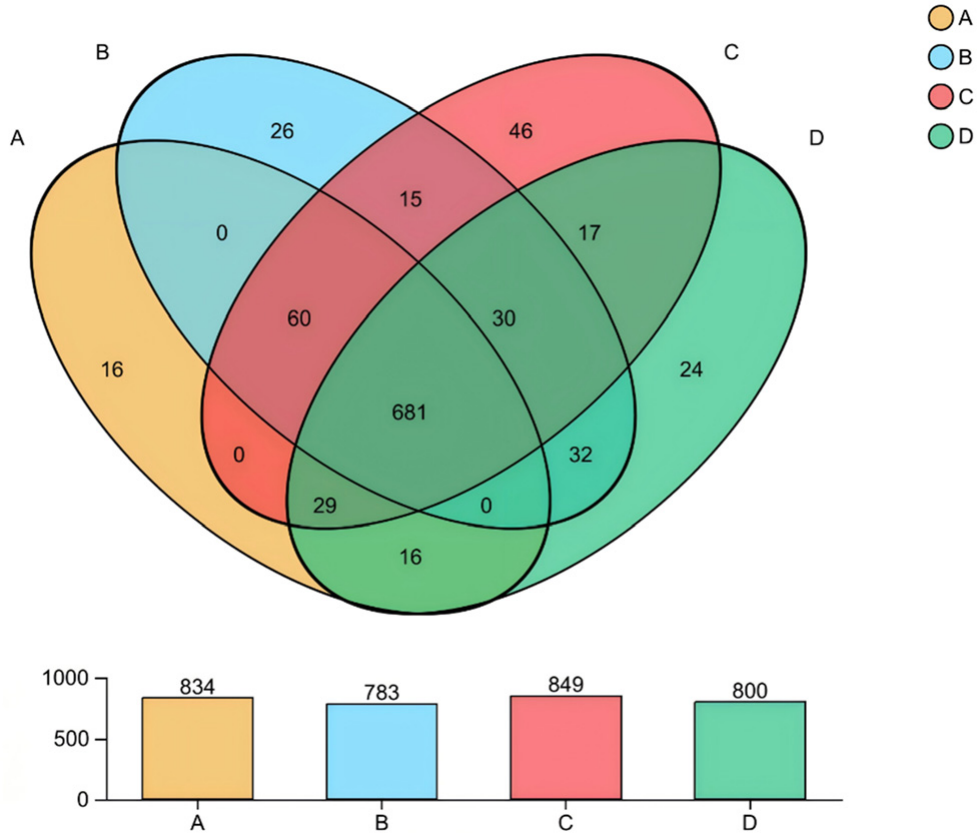
Venn plot analysis of species composition. The number of OTU taxa shared and unique to different samples is shown. The non-overlapping parts of the figure are unique to each group, and the overlapping parts are common to each group, and the numbers are marked in the corresponding range.

Venn analysis of OTU distribution revealed stratified microbial patterns among the four cohorts (Figure 2). The intersection of all groups contained 681 core OTUs (70.7 % of total OTUs) (Table 2), predominantly classified as Lachnospirales (24.5 %), Oscillospirales (20.0 %), and Bacteroidales (15.4 %), suggesting conserved microbial functions essential for gut homeostasis.

**Table 2:** Venn diagram analysis of OTU distribution in four groups of samples.

Group	A & B & C & D	B & C & D	A & C & D	C & D	B & D	A & D	D	A & B & C	B & C	A & C	C	B	A
**Num**	**649**	**30**	**32**	**17**	**32**	**16**	**24**	**31**	**15**	**29**	**46**	**26**	**16**

Elements	OTU_1; OTU_10; OTU_100; OTU_101; OTU_102; OTU_103; OTU_104; OTU_105; OTU_106; OTU_107; OTU_108; OTU_109; OTU_11; OTU_110; OTU_111; OTU_112; OTU_113; OTU_114; OTU_115; OTU_116; OTU_117; OTU_118; OTU_119; OTU_12; OTU_120; OTU_121; OTU_122; OTU_123; OTU_124; OTU_125; OTU_126; OTU_127; OTU_128; OTU_129; OTU_13; OTU_130; OTU_131; OTU_132; OTU_133; OTU_134; OTU_135; OTU_136; OTU_137; OTU_138; OTU_139; OTU_14; OTU_140; OTU_141; OTU_142; OTU_143; OTU_144; OTU_145; OTU_147; OTU_149; OTU_15; OTU_150; OTU_151; OTU_152; OTU_153; OTU_154; OTU_155; OTU_156; OTU_157; OTU_158; OTU_159; OTU_16; OTU_160; OTU_162; OTU_163; OTU_164; OTU_165; OTU_166; OTU_167; OTU_168; OTU_169; OTU_17; OTU_170; OTU_171; OTU_172; OTU_173; OTU_175; OTU_176; OTU_177; OTU_178; OTU_179; OTU_18; OTU_180; OTU_181; OTU_182; OTU_183; OTU_184; OTU_185; OTU_186; OTU_187; OTU_188; OTU_189; OTU_19; OTU_190; OTU_191; OTU_192; OTU_193; OTU_194; OTU_195; OTU_196; OTU_198; OTU_199; OTU_2; OTU_20; OTU_200; OTU_201; OTU_204; OTU_205; OTU_206; OTU_207; OTU_208; OTU_21; OTU_210; OTU_212; OTU_215; OTU_216; OTU_217; OTU_218; OTU_219; OTU_22; OTU_220; OTU_221; OTU_223; OTU_224; OTU_225; OTU_226; OTU_227; OTU_228; OTU_229; OTU_23; OTU_230; OTU_231; OTU_232; OTU_234; OTU_235; OTU_236; OTU_237; OTU_238; OTU_239; OTU_24; OTU_240; OTU_241; OTU_242; OTU_244; OTU_245; OTU_246; OTU_247; OTU_249; OTU_25; OTU_251; OTU_252; OTU_253; OTU_254; OTU_255; OTU_256; OTU_258; OTU_259; OTU_26; OTU_261; OTU_262; OTU_263; OTU_264; OTU_266; OTU_267; OTU_268; OTU_269; OTU_27; OTU_270; OTU_271; OTU_273; OTU_274; OTU_275; OTU_276; OTU_277; OTU_278; OTU_279; OTU_28; OTU_280; OTU_283; OTU_284; OTU_288; OTU_289; OTU_29; OTU_291; OTU_292; OTU_293; OTU_294; OTU_295; OTU_296; OTU_298; OTU_3; OTU_30; OTU_301; OTU_303; OTU_304; OTU_305; OTU_308; OTU_309; OTU_31; OTU_310; OTU_311; OTU_312; OTU_313; OTU_314; OTU_316; OTU_319; OTU_32; OTU_321; OTU_322; OTU_323; OTU_326; OTU_328; OTU_329; OTU_33; OTU_331; OTU_332; OTU_333; OTU_334; OTU_335; OTU_336; OTU_337; OTU_338; OTU_339; OTU_34; OTU_340; OTU_342; OTU_343; OTU_344; OTU_345; OTU_347; OTU_348; OTU_349; OTU_35; OTU_351; OTU_352; OTU_356; OTU_357; OTU_358; OTU_359; OTU_36; OTU_360; OTU_361; OTU_362; OTU_363; OTU_364; OTU_365; OTU_366; OTU_367; OTU_368; OTU_369; OTU_37; OTU_371; OTU_372; OTU_373; OTU_374; OTU_375; OTU_377; OTU_378; OTU_379; OTU_38; OTU_381; OTU_382; OTU_384; OTU_387; OTU_388; OTU_39; OTU_391; OTU_392; OTU_393; OTU_394; OTU_396; OTU_398; OTU_4; OTU_40; OTU_400; OTU_401; OTU_402; OTU_403; OTU_404; OTU_405; OTU_406; OTU_408; OTU_409; OTU_41; OTU_410; OTU_412; OTU_415; OTU_417; OTU_418; OTU_419; OTU_42; OTU_421; OTU_422; OTU_423; OTU_424; OTU_425; OTU_427; OTU_428; OTU_429; OTU_43; OTU_430; OTU_431; OTU_432; OTU_433; OTU_434; OTU_435; OTU_437; OTU_438; OTU_44; OTU_440; OTU_442; OTU_443; OTU_444; OTU_445; OTU_449; OTU_45; OTU_450; OTU_451; OTU_452; OTU_453; OTU_454; OTU_455; OTU_456; OTU_457; OTU_458; OTU_459; OTU_46; OTU_462; OTU_465; OTU_466; OTU_467; OTU_469; OTU_47; OTU_470; OTU_471; OTU_472; OTU_473; OTU_475; OTU_478; OTU_48; OTU_480; OTU_481; OTU_484; OTU_485; OTU_486; OTU_487; OTU_488; OTU_489; OTU_49; OTU_494; OTU_495; OTU_496; OTU_498; OTU_499; OTU_5; OTU_50; OTU_500; OTU_503; OTU_504; OTU_505; OTU_506; OTU_507; OTU_51; OTU_510; OTU_512; OTU_513; OTU_514; OTU_515; OTU_516; OTU_517; OTU_518; OTU_519; OTU_52; OTU_520; OTU_522; OTU_523; OTU_524; OTU_526; OTU_527; OTU_528; OTU_53; OTU_531; OTU_532; OTU_534; OTU_536; OTU_537; OTU_539; OTU_54; OTU_540; OTU_541; OTU_545; OTU_546; OTU_547; OTU_549; OTU_55; OTU_550; OTU_551; OTU_553; OTU_555; OTU_559; OTU_56; OTU_561; OTU_564; OTU_566; OTU_569; OTU_57; OTU_570; OTU_571; OTU_572; OTU_573; OTU_576; OTU_579; OTU_58; OTU_584; OTU_586; OTU_589; OTU_59; OTU_591; OTU_593; OTU_597; OTU_598; OTU_599; OTU_6; OTU_60; OTU_600; OTU_601; OTU_606; OTU_607; OTU_61; OTU_610; OTU_612; OTU_613; OTU_615; OTU_616; OTU_617; OTU_618; OTU_619; OTU_62; OTU_620; OTU_621; OTU_622; OTU_623; OTU_628; OTU_629; OTU_63; OTU_633; OTU_634; OTU_635; OTU_636; OTU_638; OTU_639; OTU_64; OTU_641; OTU_642; OTU_645; OTU_647; OTU_648; OTU_65; OTU_652; OTU_653; OTU_654; OTU_656; OTU_657; OTU_66; OTU_661; OTU_662; OTU_664; OTU_667; OTU_668; OTU_669; OTU_67; OTU_670; OTU_676; OTU_678; OTU_679; OTU_68; OTU_680; OTU_681; OTU_682; OTU_683; OTU_684; OTU_685; OTU_687; OTU_689; OTU_69; OTU_690; OTU_691; OTU_692; OTU_698; OTU_699; OTU_7; OTU_70; OTU_700; OTU_705; OTU_706; OTU_707; OTU_709; OTU_71; OTU_712; OTU_717; OTU_718; OTU_719; OTU_72; OTU_720; OTU_721; OTU_727; OTU_729; OTU_73; OTU_731; OTU_732; OTU_733; OTU_734; OTU_737; OTU_739; OTU_74; OTU_740; OTU_741; OTU_743; OTU_744; OTU_746; OTU_747; OTU_75; OTU_750; OTU_751; OTU_752; OTU_753; OTU_754; OTU_756; OTU_757; OTU_758; OTU_76; OTU_761; OTU_763; OTU_765; OTU_768; OTU_769; OTU_77; OTU_773; OTU_774; OTU_776; OTU_777; OTU_78; OTU_782; OTU_783; OTU_784; OTU_785; OTU_786; OTU_788; OTU_79; OTU_790; OTU_791; OTU_794; OTU_795; OTU_796; OTU_797; OTU_798; OTU_8; OTU_80; OTU_800; OTU_803; OTU_804; OTU_806; OTU_808; OTU_809; OTU_81; OTU_812; OTU_813; OTU_814; OTU_815; OTU_817; OTU_819; OTU_82; OTU_821; OTU_827; OTU_828; OTU_83; OTU_831; OTU_838; OTU_84; OTU_840; OTU_85; OTU_850; OTU_851; OTU_853; OTU_857; OTU_86; OTU_863; OTU_864; OTU_865; OTU_866; OTU_867; OTU_868; OTU_869; OTU_87; OTU_870; OTU_871; OTU_872; OTU_878; OTU_88; OTU_880; OTU_882; OTU_883; OTU_885; OTU_886; OTU_889; OTU_89; OTU_895; OTU_899; OTU_9; OTU_90; OTU_901; OTU_902; OTU_905; OTU_908; OTU_909; OTU_91; OTU_912; OTU_914; OTU_916; OTU_918; OTU_92; OTU_920; OTU_922; OTU_925; OTU_926; OTU_929; OTU_93; OTU_930; OTU_931; OTU_932; OTU_933; OTU_934; OTU_935; OTU_94; OTU_942; OTU_943; OTU_944; OTU_946; OTU_95; OTU_951; OTU_957; OTU_96; OTU_97; OTU_98; OTU_99	OTU_148; OTU_209; OTU_315; OTU_317; OTU_325; OTU_447; OTU_448; OTU_461; OTU_468; OTU_490; OTU_557; OTU_590; OTU_594; OTU_611; OTU_631; OTU_646; OTU_651; OTU_663; OTU_674; OTU_730; OTU_759; OTU_778; OTU_802; OTU_832; OTU_858; OTU_874; OTU_876; OTU_881; OTU_945; OTU_947	OTU_285; OTU_290; OTU_350; OTU_354; OTU_370; OTU_383; OTU_389; OTU_482; OTU_493; OTU_502; OTU_511; OTU_543; OTU_563; OTU_581; OTU_583; OTU_587; OTU_660; OTU_666; OTU_673; OTU_686; OTU_735; OTU_749; OTU_807; OTU_823; OTU_834; OTU_854; OTU_855; OTU_877; OTU_900; OTU_919; OTU_921; OTU_960	OTU_318; OTU_477; OTU_479; OTU_544; OTU_585; OTU_603; OTU_658; OTU_677; OTU_693; OTU_745; OTU_822; OTU_829; OTU_847; OTU_861; OTU_875; OTU_894; OTU_954	OTU_272; OTU_324; OTU_416; OTU_552; OTU_575; OTU_578; OTU_592; OTU_595; OTU_608; OTU_609; OTU_644; OTU_655; OTU_672; OTU_762; OTU_770; OTU_799; OTU_818; OTU_826; OTU_839; OTU_841; OTU_884; OTU_897; OTU_917; OTU_939; OTU_411; OTU_736; OTU_755; OTU_766; OTU_873; OTU_892; OTU_915; OTU_952	OTU_397; OTU_533; OTU_671; OTU_711; OTU_738; OTU_764; OTU_789; OTU_811; OTU_842; OTU_887; OTU_888; OTU_903; OTU_904; OTU_907; OTU_941; OTU_963	OTU_386; OTU_426; OTU_535; OTU_605; OTU_632; OTU_695; OTU_696; OTU_702; OTU_703; OTU_714; OTU_728; OTU_760; OTU_772; OTU_816; OTU_830; OTU_849; OTU_862; OTU_879; OTU_896; OTU_924; OTU_958; OTU_959; OTU_961; OTU_962	OTU_146; OTU_174; OTU_222; OTU_260; OTU_265; OTU_306; OTU_327; OTU_436; OTU_525; OTU_548; OTU_554; OTU_588; OTU_602; OTU_624; OTU_643; OTU_665; OTU_701; OTU_704; OTU_713; OTU_716; OTU_725; OTU_748; OTU_787; OTU_805; OTU_833; OTU_835; OTU_843; OTU_898; OTU_928; OTU_936; OTU_948	OTU_233; OTU_243; OTU_297; OTU_355; OTU_414; OTU_491; OTU_574; OTU_614; OTU_697; OTU_724; OTU_779; OTU_780; OTU_836; OTU_893; OTU_949	OTU_161; OTU_202; OTU_213; OTU_281; OTU_282; OTU_302; OTU_395; OTU_446; OTU_463; OTU_474; OTU_483; OTU_492; OTU_497; OTU_568; OTU_580; OTU_708; OTU_710; OTU_722; OTU_726; OTU_767; OTU_793; OTU_845; OTU_852; OTU_856; OTU_906; OTU_927; OTU_937; OTU_938; OTU_955	OTU_203; OTU_250; OTU_257; OTU_286; OTU_287; OTU_299; OTU_300; OTU_307; OTU_320; OTU_330; OTU_346; OTU_353; OTU_376; OTU_399; OTU_413; OTU_439; OTU_441; OTU_464; OTU_501; OTU_521; OTU_530; OTU_538; OTU_542; OTU_558; OTU_560; OTU_577; OTU_596; OTU_604; OTU_625; OTU_626; OTU_640; OTU_650; OTU_659; OTU_675; OTU_694; OTU_715; OTU_723; OTU_771; OTU_801; OTU_846; OTU_848; OTU_860; OTU_913; OTU_923; OTU_953; OTU_956	OTU_197; OTU_211; OTU_214; OTU_390; OTU_556; OTU_562; OTU_627; OTU_630; OTU_775; OTU_810; OTU_820; OTU_825; OTU_341; OTU_407; OTU_508; OTU_567; OTU_649; OTU_688; OTU_742; OTU_781; OTU_837; OTU_844; OTU_859; OTU_890; OTU_891; OTU_950	OTU_248; OTU_380; OTU_385; OTU_420; OTU_460; OTU_476; OTU_509; OTU_529; OTU_565; OTU_582; OTU_637; OTU_792; OTU_824; OTU_910; OTU_911; OTU_940

**Figure 3:**
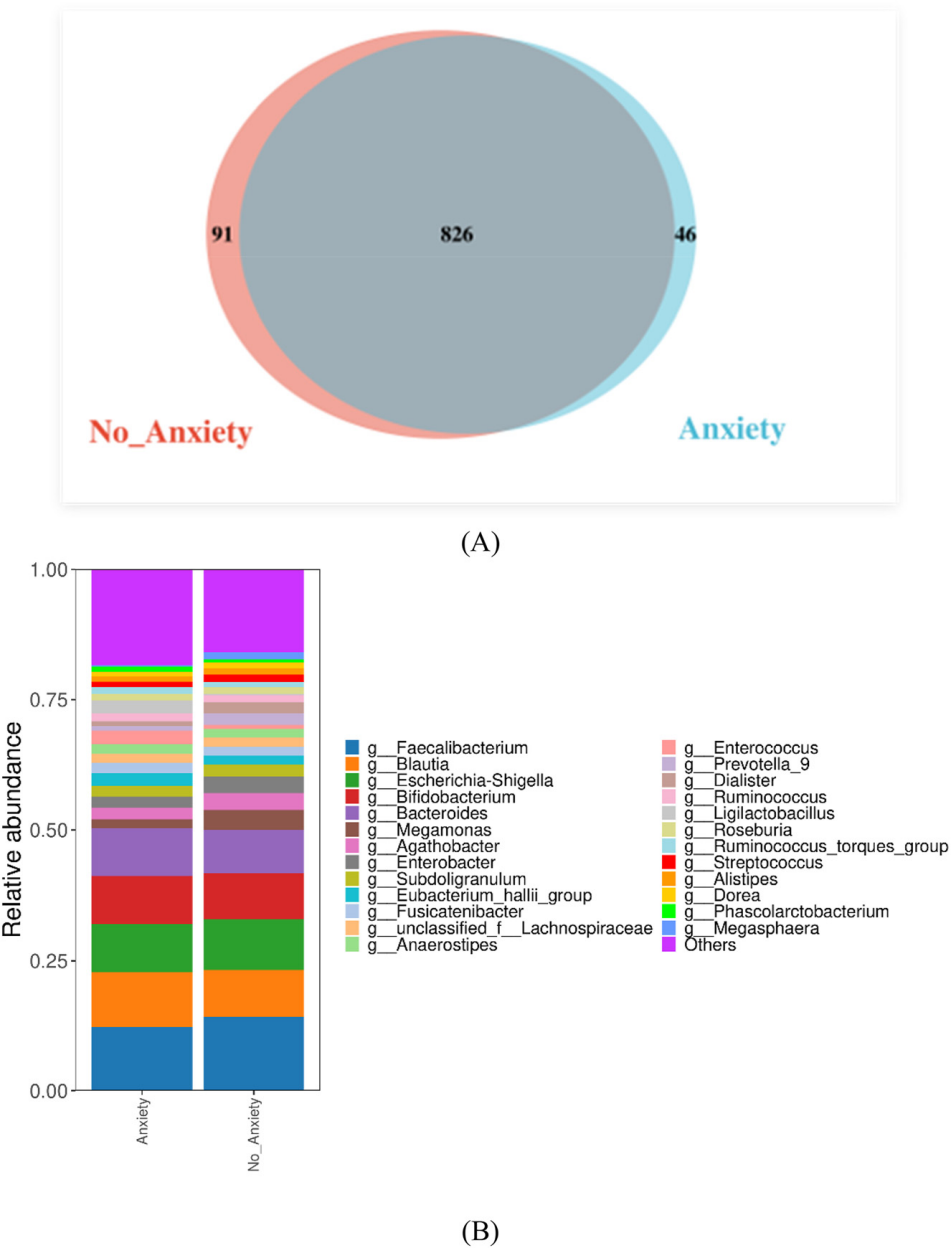

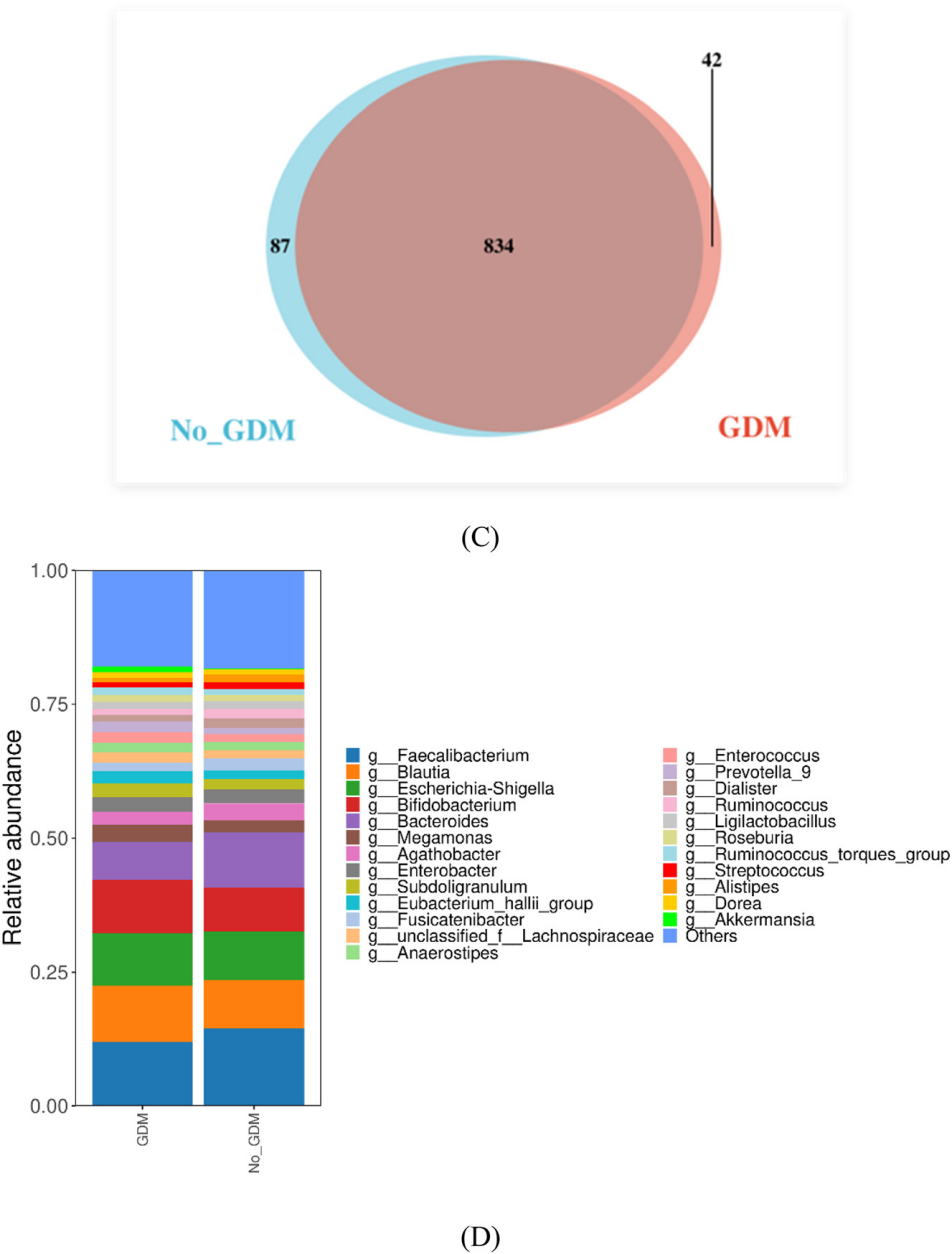
Genus-level community differences. (A) The number of OTU taxa shared and unique to A versus NA comparison groups is shown. (B) Genus-level community differences between A versus NA comparison groups. (C) The number of OTU taxa shared and unique to G versus NG comparison groups is shown. (D) Genus-level community differences between G versus NG comparison groups. For the best view, the fraction of abundance less than 1 % can be merged into other when plotting and displayed in the figure. The abscissa represents the sample, the ordinate represents the proportion of categories, and the color represents different categories.

Further analysis demonstrated differential microbial modulation by gestational diabetes mellitus (GDM) and anxiety (Figure 3): GDM-associated groups (G + A and G + NA) uniquely enriched 42 OTUs, of which 47.6 % belonged to Firmicutes, including Veillonella OTU_248, which is closely related to inflammation, and Weissella OTU_792, which is related to immune regulation. Notably, anxiety-exposed groups (NG + A and G + A) shared 32 characteristic OTUs (Bray-Curtis similarity > 0.85, FDR = 0.049), such as Lactococcus OTU_595 and Streptococcus OTU_915, which are implicated in gut-brain axis regulation.

In the dual-exposure group (G + A), we identified 26 unique OTUs not seen in the other groups, some of which were homologous to the oral microbiome by phylogenetic analysis (e.g., Fusobacterium OTU_562 and Actinomyces OTU_742), suggesting potential oral-gut axis translocation. Crucially, the healthy control group (NG + NA) contained 46 exclusive OTUs dominated by butyrate-producing bacteria, including Christensenellaceae_R-7 OTU_542. These beneficial microbes appear to safeguard gut homeostasis and modulate immunity, potentially influencing disease processes via short-chain fatty acid-mediated pathways.

### 3.3 Microbial community structure variation associated with anxiety and GDM status

We comprehensively evaluated *α*-diversity metrics across four clinically defined groups (B: GDM with anxiety, A: GDM alone, D: non-GDM with anxiety, C: healthy controls). Despite metabolic and psychological heterogeneity, all indices demonstrated remarkable stability (Supplementary Table S2–S3). The Shannon diversity index showed tight clustering across groups (A: 2.78 ± 0.66, B: 2.80 ± 0.80, C: 2.95 ± 0.74, D: 2.93 ± 0.81), with the most pronounced intergroup difference observed between groups A and D (*p* = 0.155). No significant differences were detected in: Simpson evenness index (A: 0.84 ± 0.11, B: 0.85 ± 0.11, C: 0.85 ± 0.12, D: 0.85 ± 0.14), Chao1 richness estimator (A: 274.37 ± 65.78, B: 271.05 ± 56.96, C: 282.24 ± 63.23, D: 280.79 ± 58.44) and ACE abundance-based coverage estimator (A: 272.44 ± 63.64, B: 271.90 ± 55.65, C: 283.86 ± 62.12, D: 278.21 ± 56.70). All adjusted *p*-values (p.adj) exceeded 0.4. The Pielou evenness index exhibited close clustering across groups (A: 0.51 ± 0.10, B: 0.51 ± 0.13, C: 0.54 ± 0.12, D: 0.53 ± 0.13), with the largest (albeit non-significant) difference observed between groups A and D (*p* = 0.13) (Supplementary Figure 4).

Beta-diversity analysis revealed significant restructuring of the gut microbiota associated with gestational diabetes mellitus (GDM) and anxiety status. Principal coordinates analysis (PCoA) based on Bray-Curtis dissimilarity showed that the first principal coordinate (explaining 19.78 % of variance) distinguished the microbial communities of GDM-positive groups (G + A/G + NA) from those of non-GDM groups (NG + A/NG + NA) along the clinical phenotype gradient, with tight clustering (PERMANOVA: *F* = 1.20, *R* ^2^ = 0.01, *p* = 0.216). Similarly, the microbial communities of anxiety-positive groups (G + A/NG + A) did not show significant separation from those of non-anxiety groups (G + NA/NG + NA) (PERMANOVA: *F* = 1.11, *R* ^2^ = 0.01, *p* = 0.282) (Supplementary Figures 5–6).

**Figure 4:**
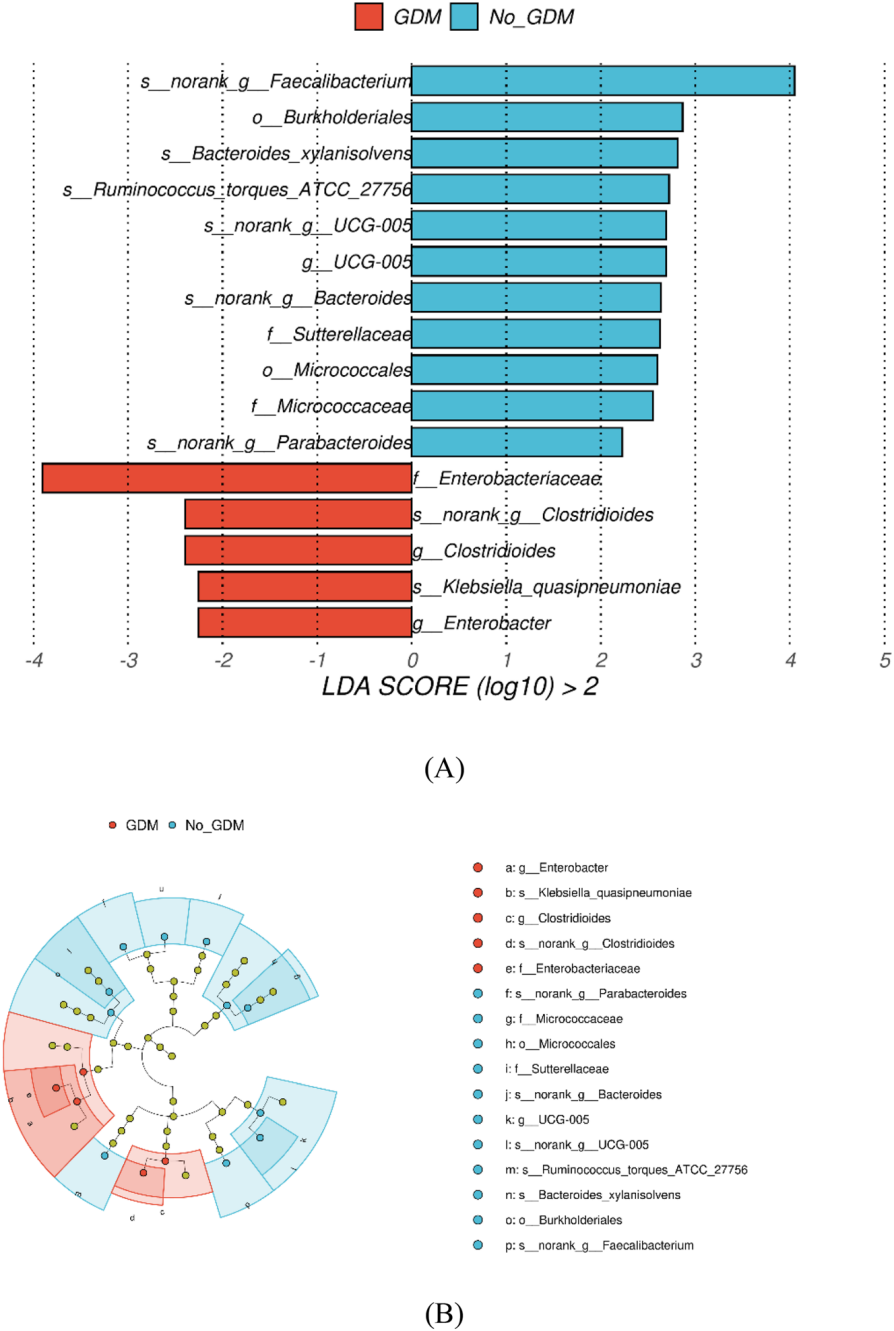

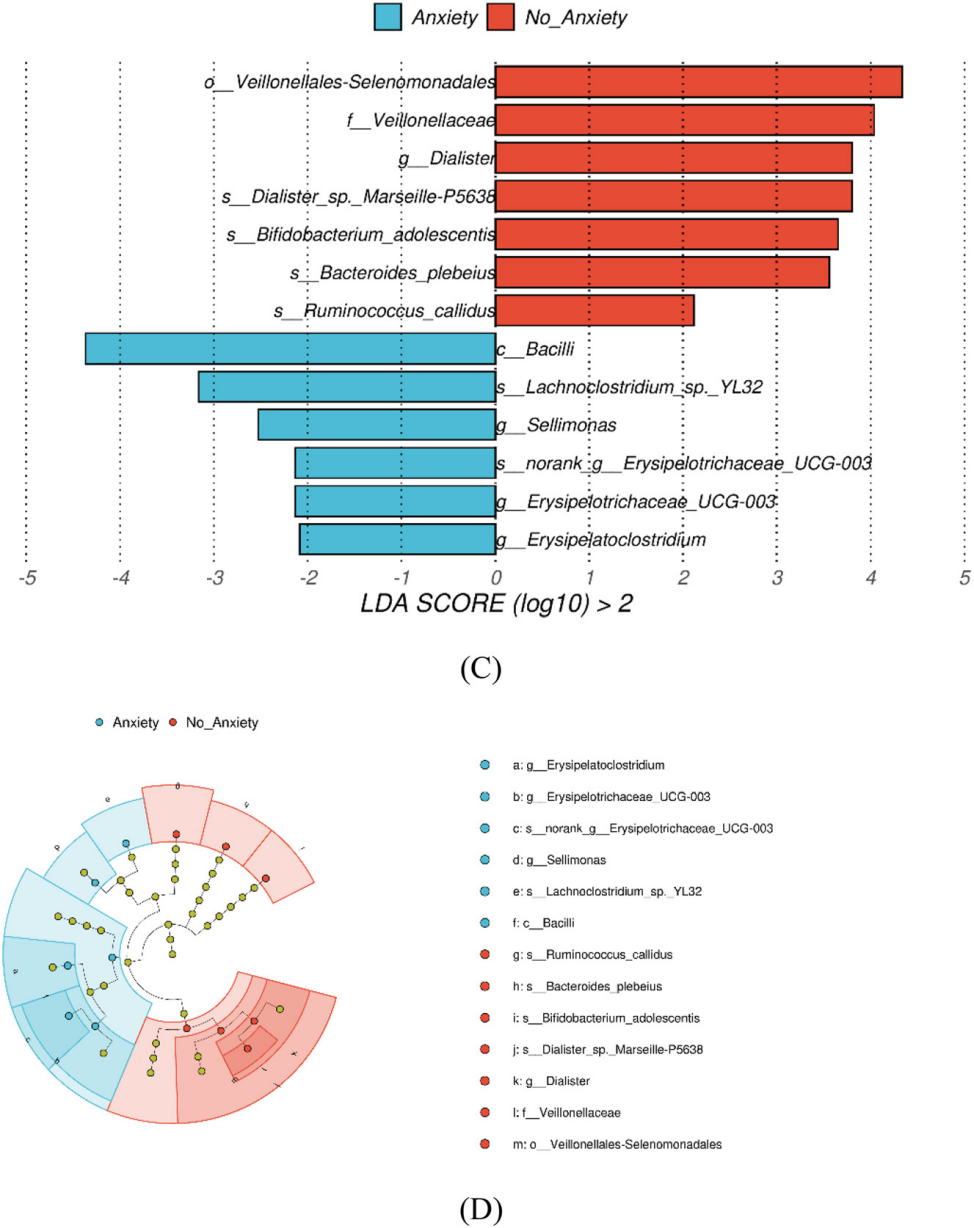
Lefse analysis bars of A versus NA and G versus NG comparison groups. The bar graph shows the significant difference Biomaker with LDA score greater than the threshold and *p*-value less than 0.05, that is, Biomaker with statistical difference, the default LDA threshold is 2.0. The color of the bar graph represents the respective group, and the length represents the LDA score, which is the degree of influence of significantly different species between groups. In the ring-shaped dendrogram, the circles radiating from inside to outside represent taxonomic levels from phylum to genus, each small circle at different taxonomic levels represents a taxon at that level, and the diameter of the small circle represents the relative abundance. Species with no significant difference were uniformly colored yellow, and the biomarker with significant difference followed the group for coloring. (A) Bar plots of LDA scores for A versus NA comparison groups. (B) Circular dendrogram of LDA score for A versus NA comparison groups. (C) Bar plots of LDA scores for G versus NG comparison groups. (D) Circular dendrogram of LDA score for G versus NG comparison groups.

A linear discriminant analysis effect size (LEFSe) biomarker analysis was performed between the GDM groups (G + A/G + NA) and the non-GDM groups (NG + A/NG + NA) (Figure 4), identifying 16 phylogenetically conserved taxonomic units (LDA > 2.0, *p* < 0.05). The GDM-specific features included enrichment of the family Enterobacteriaceae (LDA = 3.90) and depletion of the group Faecalibacterium (LDA = −4.05), with functional prediction indicating their association with the Linoleic acid metabolism pathway (KEGG KO00454, *p* = 0.0017). In the anxiety-positive groups (G + A/NG + A) compared to the non-anxiety groups (G + NA/NG + NA), the phylum Bacilli (LDA = 4.37) and the class Negativicutes (LDA = −4.33) were identified as dominant features, which may be involved in the gut-brain axis regulation through Pyruvate metabolism (KEGG K00161, *p* = 0.025) and Vitamin B6 metabolism (KEGG KO00831, *p* = 0.016). The dual-exposure group (G + A) specifically enriched the genera Ligilactobacillus (LDA = 4.08), which is of oral origin, suggesting compromised mucosal barrier integrity.

## 4 Discussion

This study revealed significant alterations in gut microbiota composition between the GDM and non-GDM groups, demonstrating a marked enrichment of Enterobacteriaceae and concurrent depletion of Faecalibacterium in the intestinal tract of GDM patients. These findings align with the observations by Sinha et al., who proposed that the second trimester in GDM is characterized by an elevated abundance of opportunistic pathogens (e.g., *Escherichia coli*) and diminished populations of short-chain fatty acid (SCFA)-producing bacteria, such as *Faecalibacterium prausnitzii*, within the gut ecosystem [14]. The expansion of Enterobacteriaceae may potentiate proinflammatory pathways, while the reduction in Ruminococcaceae (encompassing SCFA-producing taxa like *F. prausnitzii*) likely impairs microbial modulation of host insulin sensitivity. Such microbial imbalance highlights how disturbances in gut ecology can directly precipitate metabolic dysfunction central to GDM development [15], 16].

Further KEGG functional prediction analyses revealed that GDM-associated microbial consortia exhibited robust correlations with the linoleic acid metabolism pathway (KEGG KO00454). This observation echoes findings from Dan et al., who reported elevated levels of linoleic acid-derived metabolites in breast milk from GDM patients, suggesting that gut microbiota may exacerbate insulin resistance through modulation of fatty acid metabolic cascades [17]. The selective enrichment of Veillonella OTU_248 (a taxon linked to proinflammatory responses) [18] and Weissella OTU_792 (associated with immune modulation) [19] within the GDM cohort further underscores the pivotal role of gut microbial dynamics in GDM pathophysiology.

The anxiety groups (NG + A and G + A) shared 32 characteristic OTUs, with genera such as Lactococcus and Streptococcus demonstrating close associations with gut-brain axis regulation [20]. These findings align with the gut-brain axis investigations by Bravo et al., who identified that anxiety-related behaviors could induce hypothalamic-pituitary-adrenal (HPA) axis activation, subsequently driving intestinal overgrowth of Lactococcus and impairing serotonin metabolism [21]. The mild elevation in serum uric acid levels observed in anxiety groups may correlate with gut microbiota-mediated dysregulation of purine metabolism, consistent with the experimental evidence reported by Bercik et al. [22].

The LEFSe analysis revealed that the anxiety-positive group was characterized by Bacilli (LDA = 4.37) and Negativicutes (LDA = −4.33), which participate in gut-brain axis regulation through pyruvate metabolism (KEGG K00161) and vitamin B6 metabolism (KEGG KO00831). The association between dysregulated vitamin B6 metabolism and anxiety-like behaviors has been validated in multiple animal experiments [23], [24], [25], [26], suggesting that gut microbiota may influence host emotional states via neurotransmitter precursor metabolism.

The dual-exposure group (G + A) exhibited 26 unique OTUs, with the detection of oral-origin genera including Fusobacterium and Actinomyces indicating compromised mucosal barrier integrity. These observations resonate with findings by Xu et al., whose research revealed that oro-intestinal axis translocation events show significant associations with chronic inflammatory disorders [27]. The enrichment of Ligilactobacillus (LDA = 4.08) in the dual-exposure group further corroborates the oral microbial translocation hypothesis. The persistent colonization of these oral-derived bacteria may exacerbate metabolic dysregulation and anxiety symptoms in GDM patients through disruption of intestinal barrier function, a mechanism aligned with Elzayat et al.’s findings demonstrating positive correlations between gut colonization of oral taxa and anxiety scores in inflammatory bowel disease (IBD) patients [28]. The Christensenellaceae_R-7 OTU_542 unique to the healthy control group (NG + NA) may maintain intestinal homeostasis through short-chain fatty acid (SCFA) metabolism, consistent with the findings of Liu et al. and Ang et al. [29], 30].

The inability to infer causality is a fundamental limitation of this cross-sectional study, which examines the relationship between gut microbial profiles and the co-occurrence of GDM and anxiety symptoms. While our multi-omics approach identified significant microbial signatures, the temporal precedence of microbial shifts relative to clinical manifestations remains undetermined. This methodological constraint is particularly consequential in pregnancy research, where trimester-specific physiological changes (e.g., progesterone-driven immune modulation) may dynamically reshape microbial ecosystems [31]. To address this critical knowledge gap, future investigations should adopt longitudinal cohort designs with serial biospecimen collection at defined gestational windows (e.g., 8–12, 24–28, and 36–40 weeks). Such a framework would enable precise mapping of microbial succession patterns across trigestational stages and dynamic correlations between microbial trajectories and psychometric assessments (e.g., GAD-7/HAM-A scores). Recent advancements in temporal network analysis (e.g., microbial co-abundance event detection) could further elucidate how microbial consortia collectively modulate host metabolic and neuroendocrine pathways.

Sample size expansion (recommended ≥500 cases with matched controls) is imperative to enhance statistical power, particularly for detecting low-abundance but functionally pivotal taxa (relative abundance <0.01 %). Our power calculations (*α* = 0.05, *β* = 0.2) suggest current detection limits for rare operational taxonomic units (OTUs) associated with anxiety-GDM comorbidity. A larger cohort would allow stratification by GDM severity (OGTT 1 h/2 h glucose tertiles) and anxiety subtype (physical and cognitive domains), as well as robust adjustment for confounding factors including prepregnancy BMI, dietary pattern (Mediterranean diet score), and antibiotic exposure. The use of machine learning-driven identification of microbial interaction networks can predict clinical trajectories. Emerging technologies such as single-cell metagenomics and viremia analysis can further address strain-level variation and phage-bacterial interactions that may be missed by standard 16S sequencing.

In conclusion, while our findings illuminate novel microbial associations in GDM-anxiety comorbidity, their clinical implementation requires coordinated multidisciplinary efforts across epidemiology, microbial engineering, and perinatal psychiatry. By embracing longitudinal multi-omics approaches, large-scale intervention trials, and ethical precision medicine frameworks, we can transform gut microbiome research into actionable strategies for safeguarding maternal metabolic and mental health – a pressing priority in an era of escalating gestational comorbidities.

## 5 Conclusions

In conclusion, our study provides novel insights into the complex interplay between gestational diabetes mellitus (GDM), anxiety symptoms, and the gut microbiome in pregnant women. We observed distinct gut microbial signatures associated with GDM and anxiety, highlighting the potential role of gut microbiota in the pathophysiology of these conditions. The enrichment of specific bacterial taxa in GDM and anxiety groups, along with the identification of unique OTUs in the dual-exposure group, underscores the need for further exploration of microbial biomarkers for early diagnosis and intervention. The inability to establish causality is an inherent limitation of our cross-sectional design, as the temporal sequence between shifts in the microbiota and the emergence of clinical symptoms remains unresolved. Future research should employ longitudinal cohort designs with serial biospecimen collection across gestational stages to map microbial succession patterns and their correlations with psychometric assessments. Additionally, expanding the sample size and incorporating advanced technologies such as single-cell metagenomics and viremia analysis will enhance our understanding of strain-level variations and phage-bacterial interactions. By integrating multidisciplinary approaches across epidemiology, microbial engineering, and perinatal psychiatry, we can translate gut microbiome research into actionable strategies to safeguard maternal metabolic and mental health, addressing a critical priority in the context of rising gestational comorbidities.

## Supplementary Material

Supplementary Material

Supplementary Material

Supplementary Material

Supplementary Material

Supplementary Material

Supplementary Material

Supplementary Material

Supplementary Material

Supplementary Material
