# Supplementary material for: Relationship between gestational diabetes mellitus and anxiety symptoms and gut microbiome composition in pregnant women
Source: Open Life Sci. 2026 May 25;21(1):20251317. doi: 10.1515/biol-2025-1317 (PMC13201086; doi:10.1515/biol-2025-1317)
Supplement: Supplementary file 2 — Supplementary Material [file j_biol-2025-1317_suppl_002.docx]

| Supplementary Table 2 Alpha diversity index | | | | | | | | |
| --- | --- | --- | --- | --- | --- | --- | --- | --- |
| Sample | Observed | Chao1 | ACE | Shannon | Simpson | Coverage | Pielou | PD |
| A1 | 170 | 191.577 | 195.738 | 2.10114 | 0.748921 | 0.999505 | 0.409117 | 13.4291 |
| A2 | 198 | 221.625 | 219.718 | 3.12313 | 0.923361 | 0.999592 | 0.590577 | 13.937 |
| A3 | 128 | 159.538 | 173.327 | 1.22677 | 0.560055 | 0.999403 | 0.252836 | 11.0522 |
| A4 | 234 | 283.111 | 281.841 | 2.9397 | 0.891733 | 0.999242 | 0.538869 | 17.6122 |
| A5 | 281 | 304.333 | 302.408 | 2.90383 | 0.871286 | 0.999475 | 0.515014 | 18.2438 |
| A6 | 249 | 310.385 | 311.565 | 3.1472 | 0.912261 | 0.99917 | 0.570408 | 16.7971 |
| A7 | 137 | 156.895 | 160.691 | 1.67592 | 0.592173 | 0.999592 | 0.340636 | 10.123 |
| A8 | 201 | 222 | 219.993 | 3.16802 | 0.926429 | 0.999592 | 0.597367 | 13.9114 |
| A9 | 175 | 210.652 | 212.607 | 2.95697 | 0.922974 | 0.999403 | 0.572525 | 14.0609 |
| A10 | 118 | 153.15 | 156.78 | 1.4104 | 0.680037 | 0.999446 | 0.295639 | 10.5665 |
| A11 | 319 | 361.241 | 356.04 | 3.60991 | 0.939618 | 0.999271 | 0.626156 | 21.1288 |
| A12 | 227 | 272.048 | 261.615 | 2.48153 | 0.786083 | 0.999359 | 0.457429 | 15.8306 |
| A13 | 240 | 275.357 | 275.672 | 3.06739 | 0.922329 | 0.999344 | 0.559678 | 15.8037 |
| A14 | 329 | 384.194 | 379.098 | 3.4139 | 0.926785 | 0.99914 | 0.589003 | 21.627 |
| A15 | 239 | 282 | 274.074 | 3.07382 | 0.893571 | 0.999373 | 0.561278 | 16.4995 |
| A16 | 257 | 290.115 | 285.956 | 3.16023 | 0.91348 | 0.999388 | 0.569506 | 17.0427 |
| A17 | 205 | 255.324 | 263.401 | 1.63025 | 0.637929 | 0.99914 | 0.306265 | 14.3034 |
| A18 | 333 | 377.452 | 375.44 | 3.65796 | 0.942572 | 0.999228 | 0.629799 | 22.0791 |
| A19 | 242 | 258.731 | 261.029 | 3.15792 | 0.913739 | 0.999563 | 0.575325 | 15.1958 |
| A20 | 328 | 364.387 | 361.939 | 3.72042 | 0.940429 | 0.999301 | 0.642226 | 20.7306 |
| A21 | 248 | 316.056 | 297.217 | 2.64425 | 0.763021 | 0.999271 | 0.479602 | 17.427 |
| A22 | 187 | 229 | 215.78 | 2.10741 | 0.698575 | 0.999475 | 0.402861 | 13.0885 |
| A23 | 273 | 318.536 | 321.501 | 2.7958 | 0.792448 | 0.999257 | 0.498406 | 18.9192 |
| A24 | 304 | 367 | 363.663 | 3.65867 | 0.946299 | 0.999082 | 0.639961 | 20.4995 |
| A25 | 295 | 337.774 | 336.332 | 3.29027 | 0.923784 | 0.999242 | 0.578562 | 19.1351 |
| A26 | 176 | 244.9 | 242.21 | 2.1536 | 0.801988 | 0.999228 | 0.416518 | 13.9465 |
| A27 | 286 | 334.125 | 334.882 | 3.15528 | 0.893683 | 0.999184 | 0.557864 | 18.5121 |
| A28 | 193 | 228.15 | 221.286 | 2.62607 | 0.833894 | 0.999446 | 0.498997 | 15.4138 |
| A29 | 180 | 211.316 | 210.115 | 2.56196 | 0.849465 | 0.99949 | 0.493353 | 13.6562 |
| A30 | 261 | 310.043 | 301.257 | 2.92352 | 0.81793 | 0.999301 | 0.525386 | 17.9125 |
| B1 | 206 | 253.143 | 251.963 | 2.20023 | 0.789129 | 0.999344 | 0.412966 | 13.9299 |
| B2 | 325 | 360.636 | 361.777 | 3.86958 | 0.956579 | 0.999286 | 0.669035 | 20.5501 |
| B3 | 231 | 288.037 | 291.397 | 2.70228 | 0.817936 | 0.999184 | 0.496523 | 16.8395 |
| B4 | 231 | 287.4 | 273.601 | 2.92628 | 0.870074 | 0.999301 | 0.53768 | 16.0904 |
| B5 | 181 | 236.1 | 244.855 | 0.97385 | 0.406606 | 0.999155 | 0.187333 | 14.9887 |
| B6 | 249 | 292.333 | 284.407 | 3.10872 | 0.842408 | 0.999417 | 0.563434 | 15.499 |
| B7 | 341 | 398.6 | 395.535 | 3.74298 | 0.953541 | 0.999068 | 0.641813 | 21.5683 |
| B8 | 212 | 243.889 | 247.529 | 3.22068 | 0.922526 | 0.999388 | 0.601256 | 14.9703 |
| B9 | 145 | 180.652 | 188.277 | 2.26706 | 0.84511 | 0.999403 | 0.455532 | 12.0629 |
| B10 | 142 | 189.04 | 198.667 | 1.7407 | 0.736526 | 0.999286 | 0.351244 | 11.3011 |
| B11 | 236 | 276.037 | 277.143 | 2.71897 | 0.846317 | 0.999315 | 0.497631 | 16.1909 |
| B12 | 221 | 266.048 | 265.916 | 3.10174 | 0.9161 | 0.999359 | 0.574592 | 15.1572 |
| B13 | 147 | 200.261 | 201.005 | 1.58523 | 0.726795 | 0.999271 | 0.317653 | 11.2153 |
| B14 | 283 | 313.154 | 327.248 | 3.44987 | 0.938612 | 0.999286 | 0.611089 | 18.1185 |
| B15 | 324 | 352.683 | 359.848 | 3.61302 | 0.937967 | 0.999286 | 0.625009 | 20.864 |
| B16 | 203 | 250.115 | 248.82 | 2.60445 | 0.870298 | 0.999271 | 0.490185 | 14.2782 |
| B17 | 214 | 257.385 | 252.91 | 2.32088 | 0.833591 | 0.999301 | 0.432517 | 15.2912 |
| B18 | 304 | 342.077 | 340.535 | 4.0504 | 0.969657 | 0.999344 | 0.70848 | 19.3215 |
| B19 | 207 | 247.552 | 254.13 | 2.31096 | 0.824425 | 0.999286 | 0.433355 | 17.7571 |
| B20 | 245 | 264.773 | 264.831 | 3.14917 | 0.90853 | 0.999563 | 0.572445 | 16.0854 |
| B21 | 127 | 172.882 | 171 | 1.37477 | 0.646344 | 0.999417 | 0.283799 | 11.2756 |
| B22 | 285 | 305.5 | 321.134 | 3.81617 | 0.963583 | 0.999388 | 0.67513 | 18.5858 |
| B23 | 282 | 312.75 | 315.839 | 3.5753 | 0.922694 | 0.999388 | 0.633705 | 17.8585 |
| B24 | 261 | 295.731 | 293.85 | 3.11046 | 0.895301 | 0.999373 | 0.558981 | 17.5072 |
| B25 | 199 | 221.5 | 224.834 | 2.3118 | 0.785644 | 0.999475 | 0.436741 | 14.2991 |
| B26 | 203 | 229.037 | 232.472 | 2.10582 | 0.759836 | 0.999446 | 0.396337 | 14.4121 |
| B27 | 134 | 167.913 | 174.142 | 1.83495 | 0.785786 | 0.999417 | 0.374644 | 12.4877 |
| B28 | 307 | 352 | 340.266 | 3.78295 | 0.945038 | 0.999344 | 0.660563 | 22.1215 |
| B29 | 234 | 287.118 | 274.904 | 3.21315 | 0.899146 | 0.999373 | 0.588993 | 16.8591 |
| B30 | 254 | 287.3 | 278.081 | 3.3604 | 0.934054 | 0.999461 | 0.606863 | 16.3255 |
| C1 | 283 | 328.536 | 323.307 | 3.76268 | 0.960602 | 0.999257 | 0.666499 | 18.2715 |
| C2 | 282 | 307.161 | 315.451 | 3.76957 | 0.949942 | 0.999417 | 0.668138 | 18.5175 |
| C3 | 248 | 301.261 | 301.375 | 3.41064 | 0.913651 | 0.999271 | 0.618606 | 16.2614 |
| C4 | 306 | 352.406 | 354.711 | 3.37148 | 0.880144 | 0.999199 | 0.58905 | 19.0049 |
| C5 | 239 | 272 | 279.261 | 3.30633 | 0.922359 | 0.999344 | 0.603735 | 15.6424 |
| C6 | 141 | 186.042 | 189.31 | 1.32812 | 0.658106 | 0.999315 | 0.268375 | 11.8513 |
| C7 | 324 | 369 | 371.247 | 3.33797 | 0.906178 | 0.999199 | 0.577429 | 20.3276 |
| C8 | 204 | 241.625 | 247.599 | 3.32677 | 0.942062 | 0.999373 | 0.625554 | 14.0014 |
| C9 | 417 | 465.158 | 459.855 | 4.2693 | 0.971543 | 0.999111 | 0.707648 | 27.1622 |
| C10 | 237 | 271.731 | 268.285 | 2.09216 | 0.662831 | 0.999373 | 0.382615 | 15.9761 |
| C11 | 281 | 326.536 | 324.271 | 3.24072 | 0.909734 | 0.999257 | 0.574764 | 19.5976 |
| C12 | 214 | 267.261 | 271.676 | 2.34478 | 0.779986 | 0.999271 | 0.436972 | 14.6464 |
| C13 | 303 | 337.731 | 338.255 | 3.88133 | 0.956576 | 0.999373 | 0.679299 | 19.8867 |
| C14 | 238 | 282.2 | 291.865 | 2.86789 | 0.851354 | 0.999242 | 0.524078 | 18.2217 |
| C15 | 192 | 222.028 | 240.673 | 2.30961 | 0.815494 | 0.999315 | 0.439299 | 15.7877 |
| C16 | 204 | 232.5 | 239.353 | 2.9469 | 0.889943 | 0.999432 | 0.554123 | 14.3448 |
| C17 | 250 | 285.69 | 296.739 | 3.24081 | 0.921648 | 0.99933 | 0.586947 | 17.309 |
| C18 | 283 | 349.12 | 328.428 | 2.05894 | 0.65074 | 0.999155 | 0.364708 | 19.7277 |
| C19 | 261 | 281.217 | 282.468 | 3.43554 | 0.930516 | 0.999548 | 0.617401 | 16.1773 |
| C20 | 156 | 191.429 | 181.19 | 1.88281 | 0.656753 | 0.999534 | 0.372845 | 12.078 |
| C21 | 191 | 216.2 | 221.486 | 2.41402 | 0.768051 | 0.999475 | 0.459615 | 15.2769 |
| C22 | 186 | 189.75 | 189.101 | 4.10875 | 0.965177 | 0.999913 | 0.786251 | 16.7822 |
| C23 | 199 | 234.15 | 232.677 | 2.42931 | 0.821794 | 0.999446 | 0.458941 | 14.4466 |
| C24 | 151 | 211.273 | 206.768 | 1.24617 | 0.481282 | 0.999242 | 0.248375 | 12.5102 |
| C25 | 330 | 359.684 | 368.092 | 3.14143 | 0.895432 | 0.999301 | 0.54171 | 22.7562 |
| C26 | 230 | 279.111 | 282.082 | 2.85346 | 0.8858 | 0.999242 | 0.524718 | 15.6053 |
| C27 | 265 | 311.667 | 320.557 | 3.39815 | 0.941685 | 0.999184 | 0.609018 | 18.3031 |
| C28 | 199 | 226.038 | 231.011 | 2.78731 | 0.88035 | 0.999446 | 0.526572 | 13.9221 |
| C29 | 294 | 336.241 | 327.986 | 2.83513 | 0.838446 | 0.999271 | 0.498828 | 18.1257 |
| C30 | 203 | 232.333 | 230.87 | 3.09349 | 0.908256 | 0.999519 | 0.582227 | 13.0745 |
| D1 | 223 | 274.333 | 283.115 | 3.13794 | 0.925892 | 0.999184 | 0.58033 | 15.5797 |
| D2 | 271 | 337.12 | 327.92 | 3.46473 | 0.937514 | 0.999155 | 0.618468 | 18.8828 |
| D3 | 257 | 289.5 | 290.295 | 3.65078 | 0.947077 | 0.999417 | 0.657908 | 16.9578 |
| D4 | 319 | 354.357 | 350.124 | 3.68475 | 0.953424 | 0.999344 | 0.639138 | 19.5693 |
| D5 | 155 | 216.6 | 232.497 | 0.983093 | 0.483833 | 0.999184 | 0.194926 | 13.1949 |
| D6 | 242 | 287.556 | 273.564 | 3.31871 | 0.92419 | 0.999403 | 0.604618 | 16.3745 |
| D7 | 322 | 359.143 | 349.505 | 4.05487 | 0.967627 | 0.999417 | 0.702196 | 20.2841 |
| D8 | 223 | 241.056 | 239.17 | 3.35045 | 0.936942 | 0.999621 | 0.61963 | 14.9925 |
| D9 | 315 | 344.571 | 347.831 | 3.61296 | 0.934376 | 0.99933 | 0.628059 | 19.6724 |
| D10 | 307 | 338.889 | 339.912 | 3.54766 | 0.935196 | 0.999388 | 0.619479 | 18.9758 |
| D11 | 271 | 312 | 307.616 | 3.27584 | 0.892903 | 0.999388 | 0.58475 | 18.8727 |
| D12 | 259 | 322.75 | 303.229 | 3.04176 | 0.87261 | 0.999257 | 0.547391 | 16.4951 |
| D13 | 134 | 200.231 | 204.371 | 2.17543 | 0.737005 | 0.999388 | 0.444162 | 11.4149 |
| D14 | 201 | 244.24 | 249.027 | 2.92876 | 0.890342 | 0.999315 | 0.552251 | 15.1001 |
| D15 | 249 | 294.048 | 287.38 | 3.36322 | 0.913392 | 0.999359 | 0.609561 | 16.6183 |
| D16 | 298 | 319 | 322.088 | 3.70302 | 0.940428 | 0.999475 | 0.649984 | 18.3351 |
| D17 | 308 | 349.778 | 340.47 | 3.16869 | 0.898704 | 0.999301 | 0.55299 | 20.0893 |
| D18 | 262 | 295.476 | 290.47 | 3.31545 | 0.921677 | 0.999446 | 0.595411 | 18.6468 |
| D19 | 242 | 264.957 | 264.64 | 3.34042 | 0.918825 | 0.999519 | 0.608573 | 15.5425 |
| D20 | 257 | 283.64 | 282.701 | 3.08825 | 0.906846 | 0.999461 | 0.556533 | 16.6573 |
| D21 | 133 | 166.158 | 163.154 | 1.38295 | 0.646272 | 0.999475 | 0.282792 | 11.5201 |
| D22 | 324 | 383.094 | 376.498 | 2.19946 | 0.607528 | 0.999097 | 0.38048 | 20.0301 |
| D23 | 186 | 235.043 | 234.002 | 1.6208 | 0.557887 | 0.999301 | 0.310156 | 14.2086 |
| D24 | 183 | 210.391 | 215.179 | 2.86219 | 0.889143 | 0.999475 | 0.549418 | 12.6716 |
| D25 | 311 | 342.138 | 343.931 | 3.51514 | 0.916411 | 0.999373 | 0.612416 | 18.9156 |
| D26 | 137 | 243.071 | 219.145 | 1.13627 | 0.524184 | 0.999199 | 0.230949 | 12.2717 |
| D27 | 106 | 145.375 | 143.163 | 1.63186 | 0.741114 | 0.999475 | 0.349926 | 9.63262 |
| D28 | 208 | 251 | 254.671 | 3.22359 | 0.926167 | 0.999359 | 0.603947 | 13.6696 |
| D29 | 242 | 281.048 | 274.504 | 3.13751 | 0.898263 | 0.999403 | 0.571607 | 15.7311 |
| D30 | 194 | 237 | 236.052 | 2.86303 | 0.850391 | 0.999373 | 0.543489 | 13.3706 |
