# Supplementary material for: Relationship between gestational diabetes mellitus and anxiety symptoms and gut microbiome composition in pregnant women
Source: Open Life Sci. 2026 May 25;21(1):20251317. doi: 10.1515/biol-2025-1317 (PMC13201086; doi:10.1515/biol-2025-1317)
Supplement: Supplementary file 3 — Supplementary Material [file j_biol-2025-1317_suppl_003.docx]

| Supplementary Table 3 Significant differences in alpha diversity index | | | | | | | | | | | |
| --- | --- | --- | --- | --- | --- | --- | --- | --- | --- | --- | --- |
| .y. | group1 | group2 | n1 | n2 | statistic | p | p.signif | p.adj | p.adj.signif | method | p.adjust.method |
| ACE | B | A | 30 | 30 | 443 | 0.924 | ns | 0.924 | ns | Wilcoxon | fdr |
| Chao1 | B | A | 30 | 30 | 433 | 0.809 | ns | 0.809 | ns | Wilcoxon | fdr |
| Observed | B | A | 30 | 30 | 444 | 0.935 | ns | 0.935 | ns | Wilcoxon | fdr |
| Pielou | B | A | 30 | 30 | 472 | 0.752 | ns | 0.752 | ns | Wilcoxon | fdr |
| Shannon | B | A | 30 | 30 | 472 | 0.752 | ns | 0.752 | ns | Wilcoxon | fdr |
| Simpson | B | A | 30 | 30 | 476 | 0.708 | ns | 0.708 | ns | Wilcoxon | fdr |
| ACE | A | C | 30 | 30 | 410 | 0.562 | ns | 0.562 | ns | Wilcoxon | fdr |
| Chao1 | A | C | 30 | 30 | 437 | 0.854 | ns | 0.854 | ns | Wilcoxon | fdr |
| Observed | A | C | 30 | 30 | 417 | 0.631 | ns | 0.631 | ns | Wilcoxon | fdr |
| Pielou | A | C | 30 | 30 | 383 | 0.328 | ns | 0.328 | ns | Wilcoxon | fdr |
| Shannon | A | C | 30 | 30 | 385 | 0.343 | ns | 0.343 | ns | Wilcoxon | fdr |
| Simpson | A | C | 30 | 30 | 412 | 0.582 | ns | 0.582 | ns | Wilcoxon | fdr |
| ACE | D | A | 30 | 30 | 479 | 0.676 | ns | 0.676 | ns | Wilcoxon | fdr |
| Chao1 | D | A | 30 | 30 | 477 | 0.697 | ns | 0.697 | ns | Wilcoxon | fdr |
| Observed | D | A | 30 | 30 | 478.5 | 0.679 | ns | 0.679 | ns | Wilcoxon | fdr |
| Pielou | D | A | 30 | 30 | 553 | 0.13 | ns | 0.13 | ns | Wilcoxon | fdr |
| Shannon | D | A | 30 | 30 | 547 | 0.155 | ns | 0.155 | ns | Wilcoxon | fdr |
| Simpson | D | A | 30 | 30 | 507 | 0.406 | ns | 0.406 | ns | Wilcoxon | fdr |
| ACE | B | C | 30 | 30 | 405 | 0.513 | ns | 0.513 | ns | Wilcoxon | fdr |
| Chao1 | B | C | 30 | 30 | 421 | 0.676 | ns | 0.676 | ns | Wilcoxon | fdr |
| Observed | B | C | 30 | 30 | 414 | 0.6 | ns | 0.6 | ns | Wilcoxon | fdr |
| Pielou | B | C | 30 | 30 | 406 | 0.523 | ns | 0.523 | ns | Wilcoxon | fdr |
| Shannon | B | C | 30 | 30 | 404 | 0.504 | ns | 0.504 | ns | Wilcoxon | fdr |
| Simpson | B | C | 30 | 30 | 432 | 0.797 | ns | 0.797 | ns | Wilcoxon | fdr |
| ACE | B | D | 30 | 30 | 411 | 0.572 | ns | 0.572 | ns | Wilcoxon | fdr |
| Chao1 | B | D | 30 | 30 | 407 | 0.532 | ns | 0.532 | ns | Wilcoxon | fdr |
| Observed | B | D | 30 | 30 | 410 | 0.559 | ns | 0.559 | ns | Wilcoxon | fdr |
| Pielou | B | D | 30 | 30 | 404 | 0.504 | ns | 0.504 | ns | Wilcoxon | fdr |
| Shannon | B | D | 30 | 30 | 404 | 0.504 | ns | 0.504 | ns | Wilcoxon | fdr |
| Simpson | B | D | 30 | 30 | 414 | 0.602 | ns | 0.602 | ns | Wilcoxon | fdr |
| ACE | D | C | 30 | 30 | 448 | 0.982 | ns | 0.982 | ns | Wilcoxon | fdr |
| Chao1 | D | C | 30 | 30 | 474 | 0.73 | ns | 0.73 | ns | Wilcoxon | fdr |
| Observed | D | C | 30 | 30 | 450 | 1 | ns | 1 | ns | Wilcoxon | fdr |
| Pielou | D | C | 30 | 30 | 478 | 0.686 | ns | 0.686 | ns | Wilcoxon | fdr |
| Shannon | D | C | 30 | 30 | 465 | 0.832 | ns | 0.832 | ns | Wilcoxon | fdr |
| Simpson | D | C | 30 | 30 | 470 | 0.775 | ns | 0.775 | ns | Wilcoxon | fdr |
| ACE | GDM | No_GDM | 60 | 60 | 1647 | 0.423 | ns | 0.423 | ns | Wilcoxon | fdr |
| Chao1 | GDM | No_GDM | 60 | 60 | 1688 | 0.558 | ns | 0.558 | ns | Wilcoxon | fdr |
| Observed | GDM | No_GDM | 60 | 60 | 1662.5 | 0.472 | ns | 0.472 | ns | Wilcoxon | fdr |
| Pielou | GDM | No_GDM | 60 | 60 | 1540 | 0.173 | ns | 0.173 | ns | Wilcoxon | fdr |
| Shannon | GDM | No_GDM | 60 | 60 | 1546 | 0.183 | ns | 0.183 | ns | Wilcoxon | fdr |
| Simpson | GDM | No_GDM | 60 | 60 | 1651 | 0.436 | ns | 0.436 | ns | Wilcoxon | fdr |
| ACE | Anxiety | No_Anxiety | 60 | 60 | 1775 | 0.898 | ns | 0.898 | ns | Wilcoxon | fdr |
| Chao1 | Anxiety | No_Anxiety | 60 | 60 | 1805 | 0.981 | ns | 0.981 | ns | Wilcoxon | fdr |
| Observed | Anxiety | No_Anxiety | 60 | 60 | 1786.5 | 0.946 | ns | 0.946 | ns | Wilcoxon | fdr |
| Pielou | Anxiety | No_Anxiety | 60 | 60 | 1909 | 0.569 | ns | 0.569 | ns | Wilcoxon | fdr |
| Shannon | Anxiety | No_Anxiety | 60 | 60 | 1888 | 0.646 | ns | 0.646 | ns | Wilcoxon | fdr |
| Simpson | Anxiety | No_Anxiety | 60 | 60 | 1885 | 0.657 | ns | 0.657 | ns | Wilcoxon | fdr |
