# Supplementary figures and images for: Relationship between gestational diabetes mellitus and anxiety symptoms and gut microbiome composition in pregnant women
Source: Open Life Sci. 2026 May 25;21(1):20251317. doi: 10.1515/biol-2025-1317 (PMC13201086; doi:10.1515/biol-2025-1317)

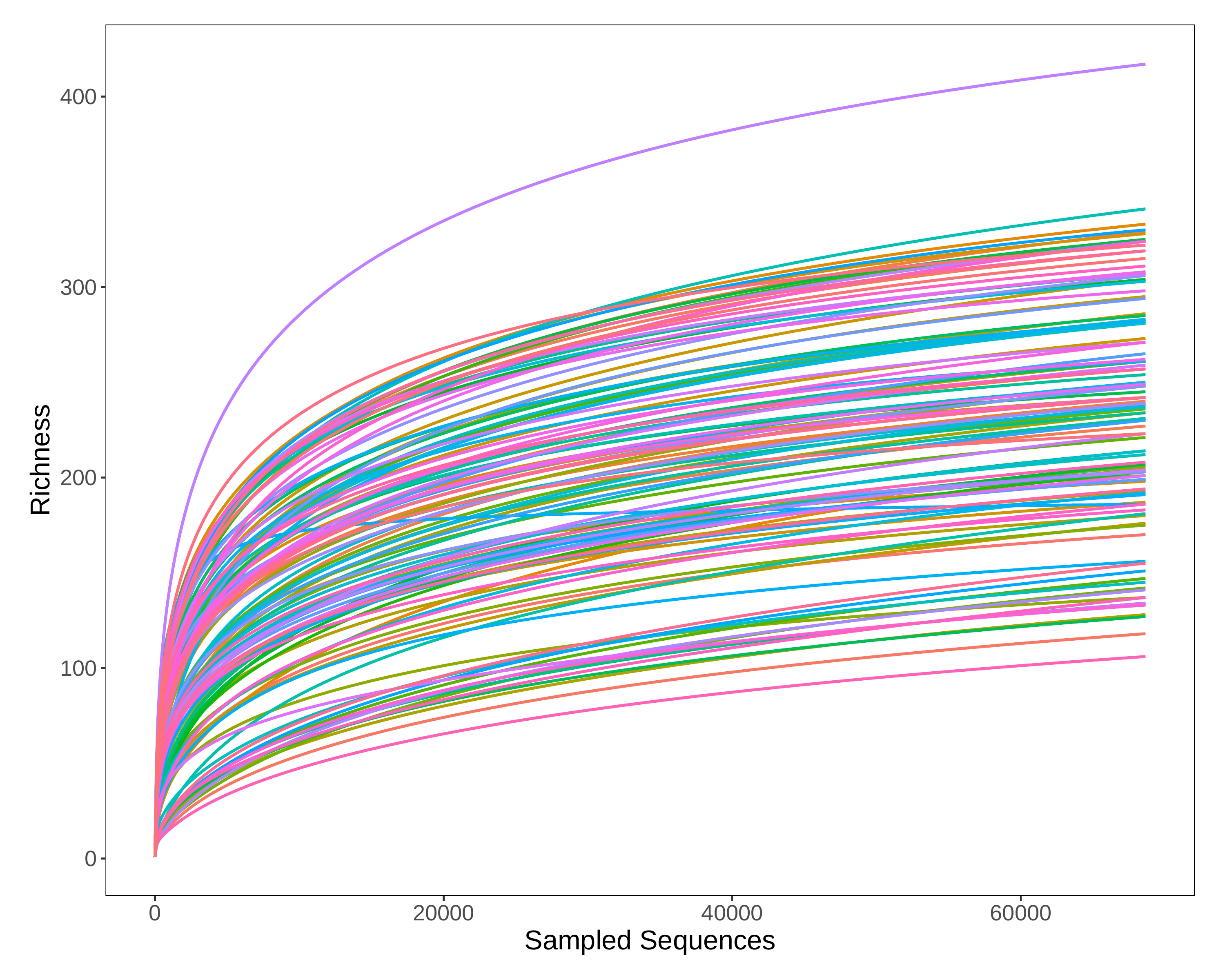

Supplement: Supplementary file 4 — Supplementary Material [file j_biol-2025-1317_suppl_004.png]

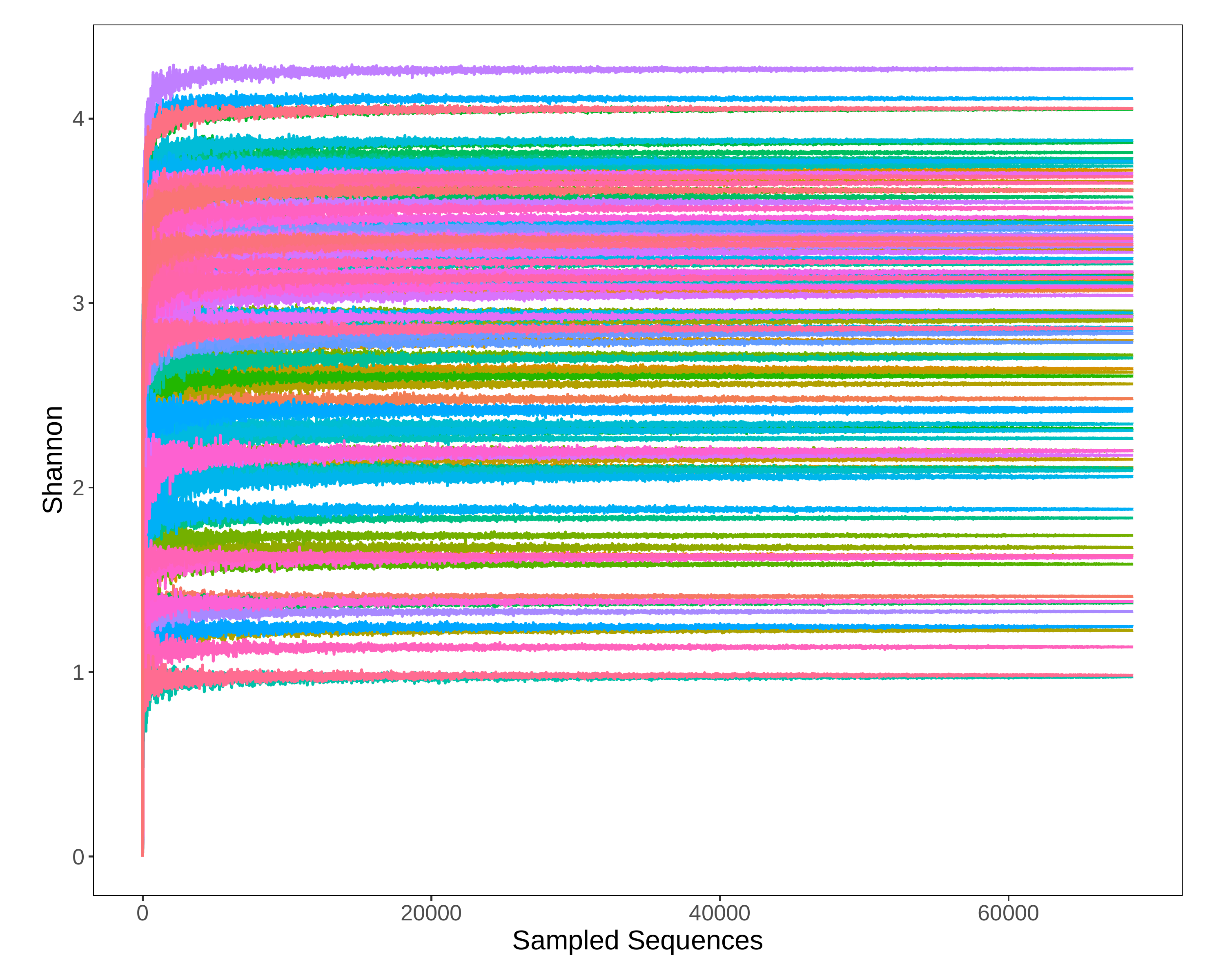

Supplement: Supplementary file 5 — Supplementary Material [file j_biol-2025-1317_suppl_005.png]

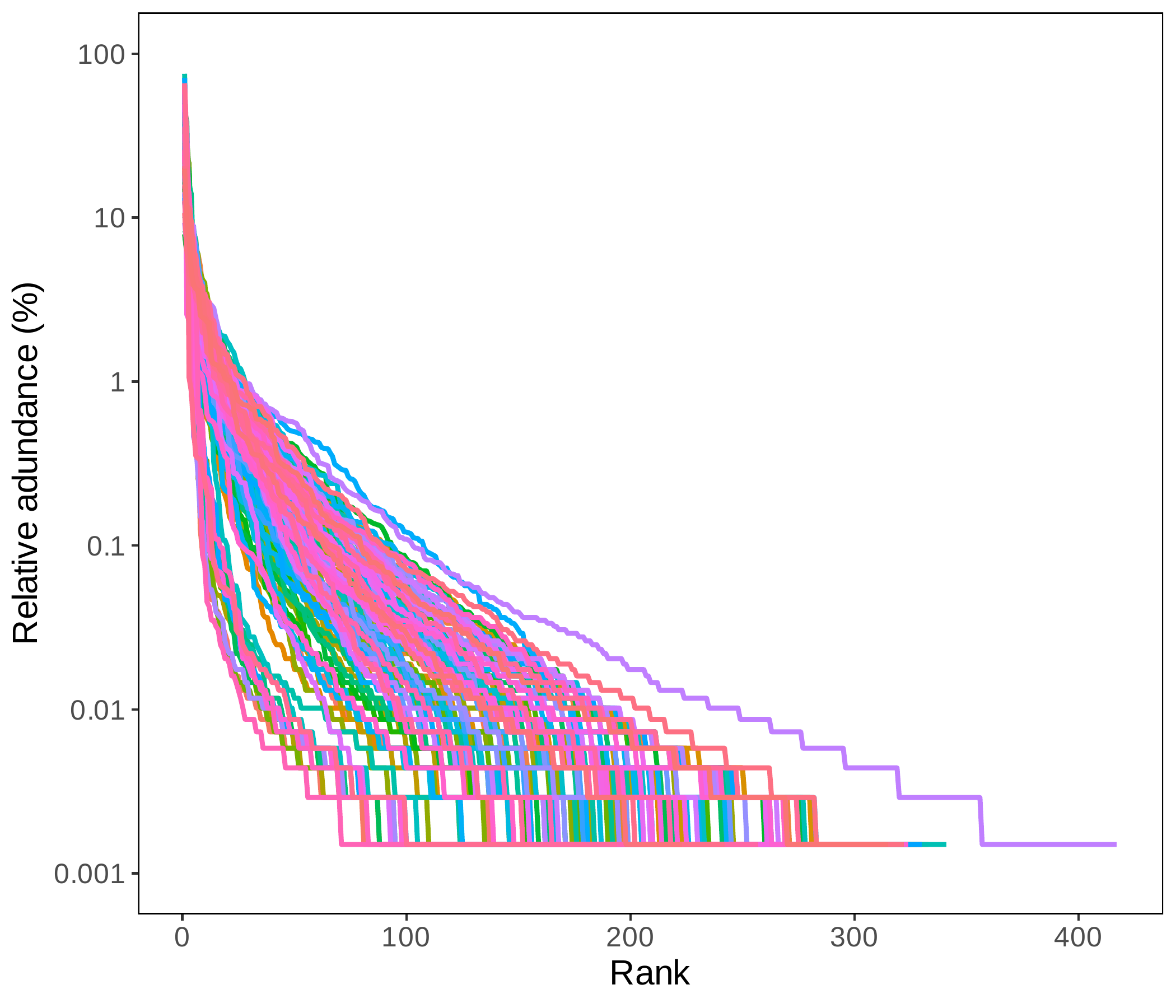

Supplement: Supplementary file 6 — Supplementary Material [file j_biol-2025-1317_suppl_006.png]

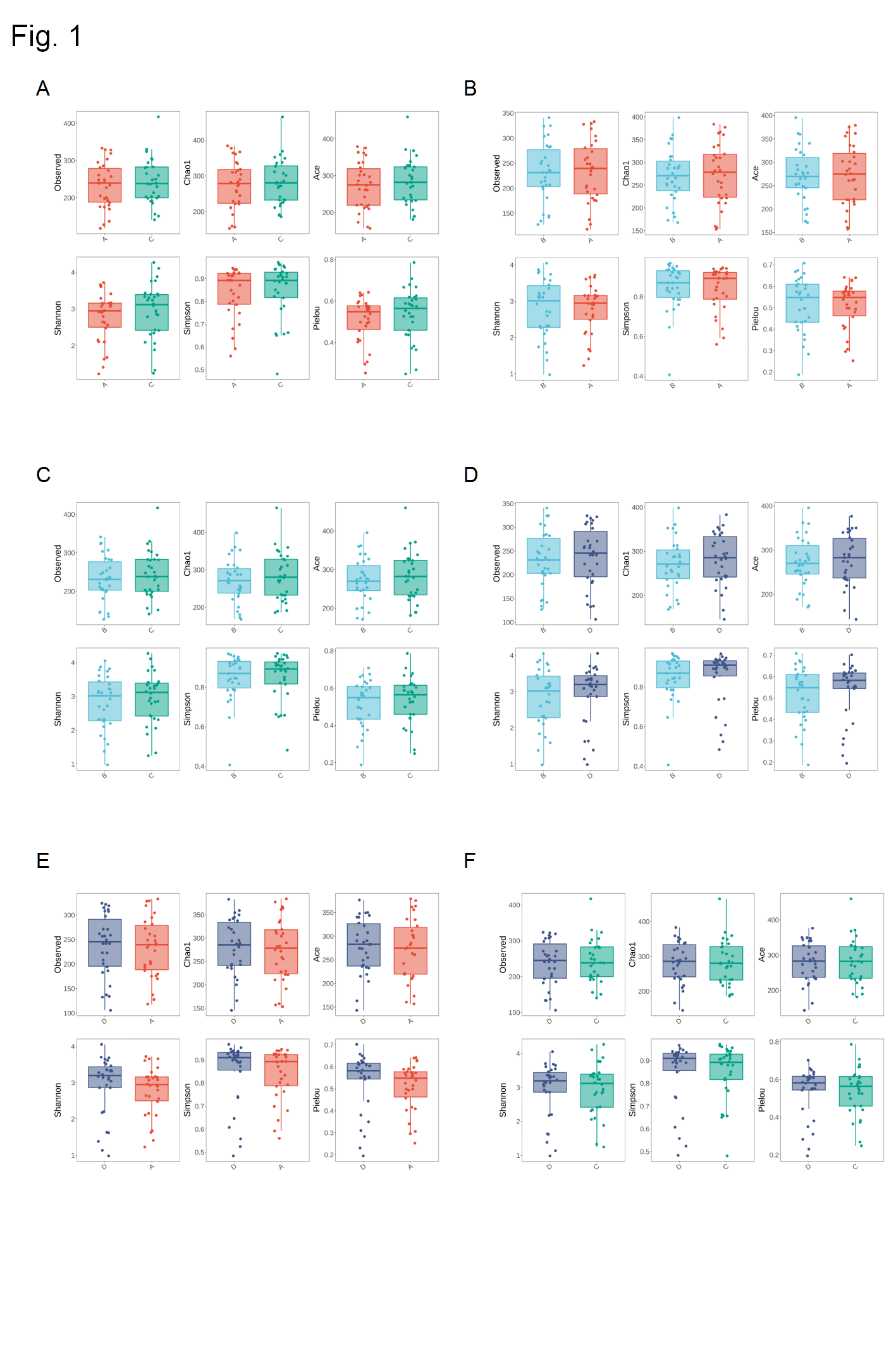

Supplement: Supplementary file 7 — Supplementary Material [file j_biol-2025-1317_suppl_007.png]

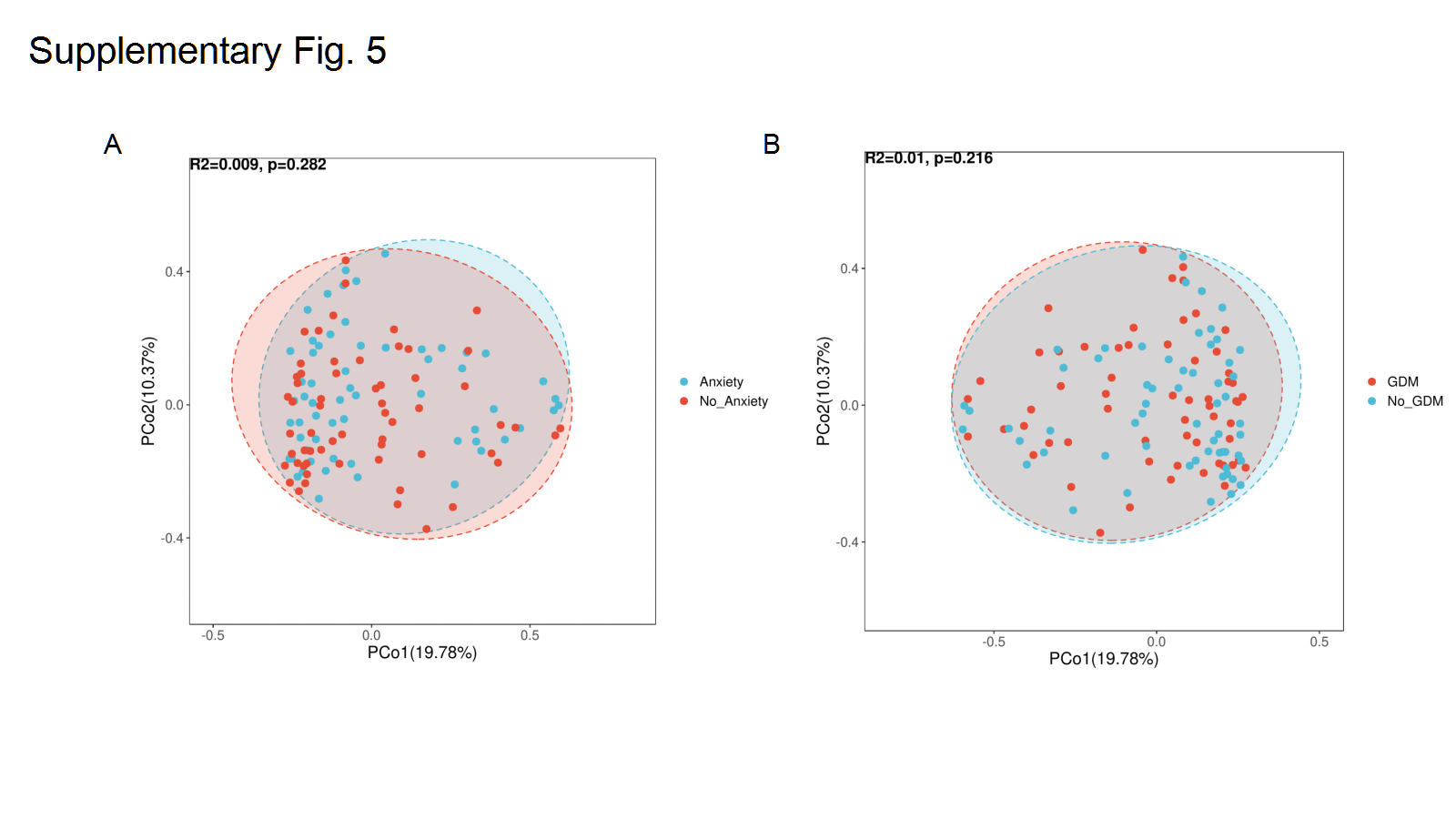

Supplement: Supplementary file 8 — Supplementary Material [file j_biol-2025-1317_suppl_008.png]

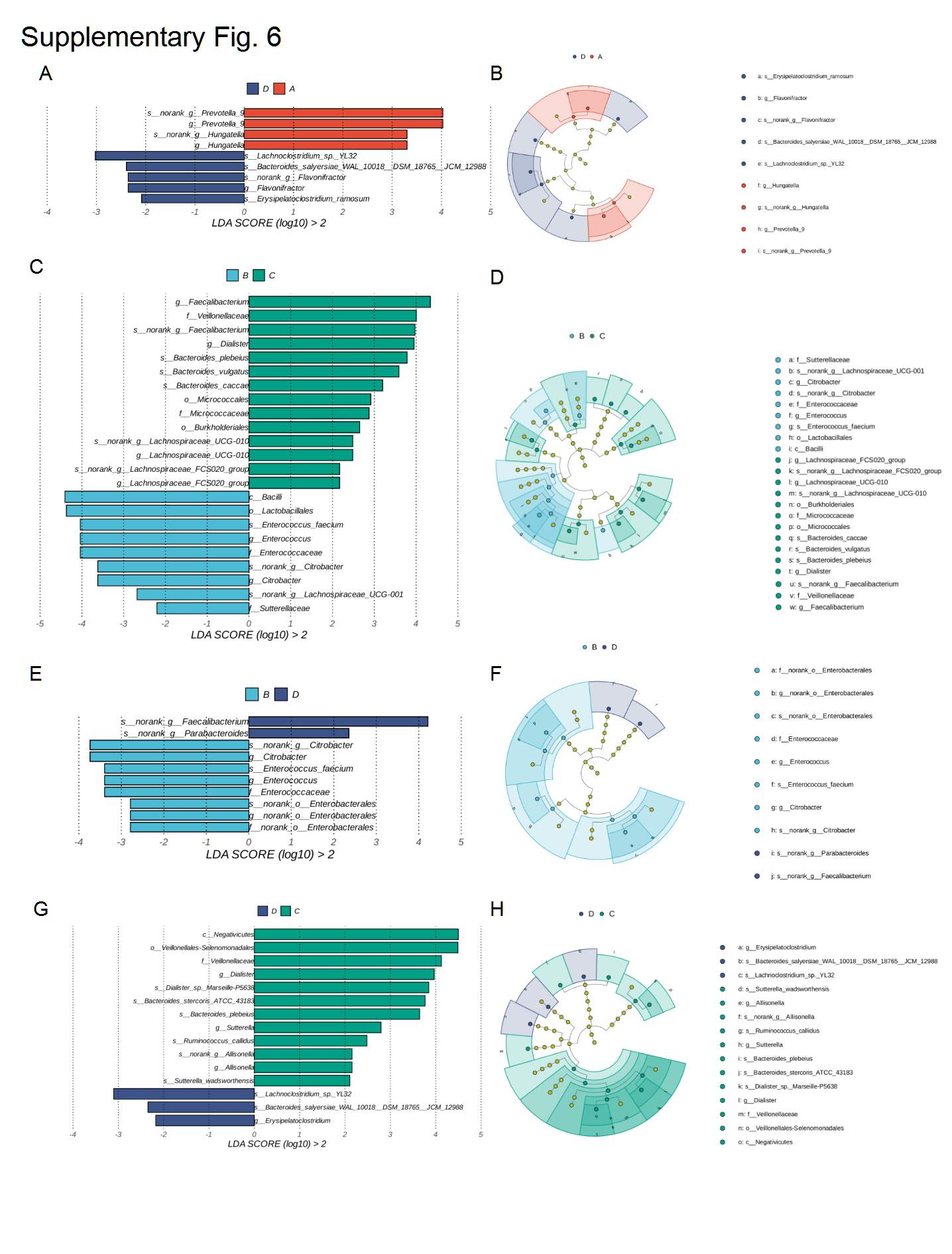

Supplement: Supplementary file 9 — Supplementary Material [file j_biol-2025-1317_suppl_009.png]
